# Synthesis of novel 3-hydroxy-2-naphthoic hydrazones as selective chemosensors for cyanide ions†

**DOI:** 10.1039/d3ra00788j

**Published:** 2023-05-18

**Authors:** Rima D. Alharthy, Ifra Urooj, Mussarat Tasleem, Muhammad Khalid, Muhammad Adnan Asghar, Shaista Ijaz Khan, Muhammad Ajmal, Nadeem Ahmed, Zahid Shafiq

**Affiliations:** a Department of Chemistry, Science & Arts College, King Abdulaziz University Rabigh Branch Rabigh 21911 Saudi Arabia; b Institute of Chemical Sciences, Bahauddin Zakariya University 60800 Multan Pakistan zahidshafiq@bzu.edu.pk; c Institute of Chemistry, Khwaja Fareed University of Engineering & Information Technology Rahim Yar Khan 64200 Pakistan khalidhej@hotmail.com Khalid@iq.usp.br; d Centre for Theoretical and Computational Research, Khwaja Fareed University of Engineering & Information Technology Rahim Yar Khan 64200 Pakistan; e Department of Chemistry, Division of Science and Technology, University of Education Lahore Pakistan

## Abstract

The development of an effective and selective chemosensor for CN^−^ ions has become the need of the hour due to their hazardous impact on the environment and humans. Herein, we report the synthesis of two novel chemosensors, IF-1 and IF-2 based on 3-hydroxy-2-naphthohydrazide and aldehyde derivatives that have shown selective sensing of CN^−^ ions. IF-2 exhibited exclusive binding with CN^−^ ions that is further confirmed by the binding constant value of 4.77 × 10^4^ M^−1^ with a low detection limit (8.2 μM). The chemosensory potential is attributed to deprotonation of the labile Schiff base center by CN^−^ ions that results in a color change from colorless to yellow as visible by the naked eye. Accompanying this, a DFT study was also performed in order to find the interaction between the sensor (IF-1) and its ions (F^−^). A notable charge transfer from 3-hydroxy-2-naphthamide to 2,4-di-*tert*-butyl-6-methylphenol, was indicated by the FMO analysis. The QTAIM analysis revealed that in the complex compound, the strongest pure hydrogen–hydrogen bonding was observed between H53 and H58, indicated by a *ρ* value of +0.017807. Due to its selective response, IF-2 can be successfully used for making test strips for the detection of CN^−^ ions.

## Abstract 2

The development of an effective and selective chemosensor for CN^−^ ions has become the need of the hour due to their hazardous impact on the environment and humans.

## Graphical abstract

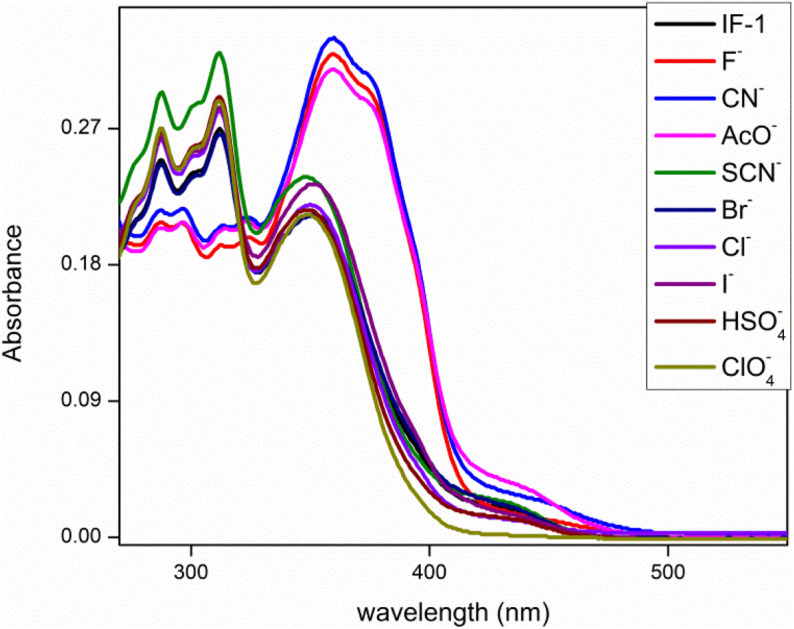

## 1. Introduction

Designing a molecular device that is capable of binding a selective analyte in solution and making a change in the form of color or photoemission has opened routes for advancing research on supramolecular chemistry for chemical sensing.^1^ Supramolecular chemistry provides a kind of host–guest compatibility between chemosensor and analyte that bind with each other through weak linkages like π–π interactions, van der Waals, H-bonding, weak polar interactions, *etc.*^2^ Molecular recognition actions of both host and guest provide a selective response that is different from other previously designed chemosensors and possesses unique, optical, biological, magnetic and catalytic properties.^3^ In standard protocol, binding site (host) and signal subunit (guest) species bind through covalent interactions making an irreversible complex, while in the supramolecular approach a host–guest complex is formed *via* weak interactions that do not induce permanent change.^4^ Moreover controlled reaction conditions may give an effective binding ability and equilibrium position can also be controlled.^5^

With increasing human demands more advancement in industry and technology is needed that is putting the human health and environment at risk.^6^ With growing technology and industries, pollution is increasing on daily bases.^7^ Anions are equally important as well as hazardous if used in excess. As far as we are concerned CN^−^ is commonly known ion having a very widespread use both in domestic and industrial life.^8,9^ They are verified as dangerous ion for human as their intake and absorption in body is quite higher than expelling them out that causes them to form aggregates and clusters causing anomalies.^10,11^

CN^−^ ions are popular in industry due to their widespread use in gold extraction, tanning, metallurgical operations, fiber synthesis and electroplating.^12^ Cyanide gas is known to have its application as pest and vermin killer in buildings, boats *etc.* and as warfare agent.^13^ Despite of its tremendous use CN^−^ possess a very high toxicity level, even they can lead to death of a person when used above recommended range that is 0.05 mg kg^−1^ body weight.^14^ CN^−^ ions binds with Fe^3+^ in metalloenzyme and heme group in body and hence retarding the metabolic process as cellular respiration, blocking the activities of cells and hence leading to death within mins.^15^ Moreover CN^−^ ions can be ingested through lens, skin and respiratory tract causing vascular necrosis, breathing disorders, unconsciousness, nervous system breakdown and many other physical disorders.^16,17^ Due to such poisonous effects various methods has been used to detect this ion that include electrochemical methods,^18^ titrimetry,^19^ voltammetry,^20,21^ chromatographic separations.^22^ These methods have some drawbacks such as time consuming, expensive instrument is required and not easy to handle. Hence there is a need to design methods that are economic, easy to use and give a very quick response. For that purpose chemosensor are suitable as they are easy to handle and less expensive instrument is required. Also they have advantage of naked eyes view.^23^

Chemosensors are application of supramolecular chemistry known for their quick and selective response, sensitivity, a detectable visual signal and easy instrumentation.^24^ In this way we can detect the desired ions within few seconds with excellent results.^25^ These results and visual response vary depending upon the structural and chemical properties of each chemosensors that is dependent upon H-bonding framework, nucleophilic addition and chelation of anion and receptor.^26^ Based on their framework different chemosensors have been synthesized till date such as amide, polyalcohols, urea, thiourea, azacrown, porphyrin based, binaphthol, fused ring heterocycles, metal complexes, polyamide, hydrazaones, indole chemosensors having different applications.^27^ Using these features we have synthesized two aromatic Schiff base based chemosensors IF-1 and IF-2 using hydrazide and aldehyde that have shown a very selective response towards CN^−^ ion even when F^−^ is also present in the solution.

## 2. Materials and methods

All the solvents and reagents used for synthesis were of analytical grade that were purchased from Oakwood Chemicals and Sigma Aldrich. The reaction was carried out under standard conditions using oven-dried round bottom flask. Progress of the reaction was monitored by TLC using silica-gel based thin layers in a solvent ratio 1 : 3 (ethyl acetate: petroleum ether). All the ions such as fluoride (F^−^), bromide (Br^−^), acetate (AcO^−^), chloride (Cl^−^), cyanide (CN^−^), perchlorate, and bisulfate in the form of tetra butyl ammonium salts were purchased from Sigma Aldrich. The structure of synthesized chemosensors IF-1 and 1F-2 were confirmed using ^1^H-NMR, HRMS and UV-visible characterization techniques. Solvents used for analysis were of spectroscopic grade. ^1^H-NMR and ^13^C NMR spectra were obtained at frequencies 300 MHz and 100 MHz respectively in DMSO-*d*_6_ solvent where TMS was used as internal standard. The absorption and transmission studies of light (UV-vis spectroscopy) were recorded using Shimadzu UV-1800 spectrophotometer.

### 2.1. Computational procedure

For current study, Gaussian 09 program^28^ was utilized to understand the development of interaction between chemosensor (IF-1) and its ion (fluoride). For this purpose, optimization of sensor and its complex (formed by the development of interaction between IF-1 and F^−^) was accomplished at M06/6-311G (d,p)^29^ functional of DFT through restricted method to obtain true minima structures. From these optimized geometries, further frontier molecular orbitals (FMOs), global reactivity parameters (GRPs), molecular electrostatic potential (MEP) and quantum theory of atoms in molecules (QTAIM) analyses were performed to elucidate the charge transference and development of interaction between neutral sensor and its ion. Various softwares like Avogadro,^30^ Multiwfn 3.7 (ref. 31) and Gauss View 5.0 (ref. 32) were utilized to interpret the data from outputs.

### 2.2. Synthesis

**Scheme 1:**
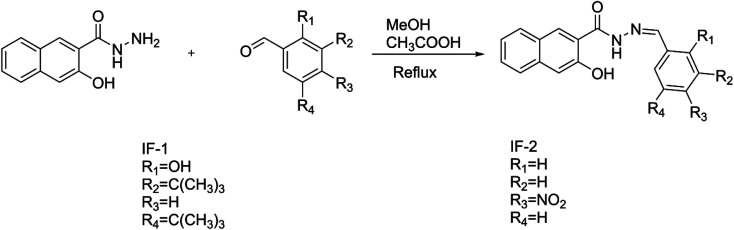
Synthesis of chemosensor (IF-1 and IF-2).

A single step condensation reaction was carried out to synthesize IF-1 and IF-2. Respective aldehyde (3,5-di-*tert*-butyl-2-hydroxybenzaldehyde (0.1 g, 0.42 mmol) and 4-nitrobenzaldehyde (0.1 g, 0.66 mmol)) were dissolved in 15 ml methanol and stirred for 20 min. Then 3-hydroxy-2-naphthohydrazide (0.42 mmol and 0.66 mmol) was added. A catalytic amount (2–3 drops) of acetic acid was added into the reaction. The reaction was allowed to proceed at constant temperature under reflux for 4–5 hours after some time solid precipitates. After the completion of reaction as confirmed by TLC, precipitates were filtered off and washed with methanol. Precipitates obtained were dried and subjected to characterization SI-1-6. Following Scheme 1 illustrates the reaction mechanism of chemosensors obtained; (*E*)-*N*′-(3,5-di-*tert*-butyl-2-hydroxybenzylidene)-3-hydroxy-2-naphthohydrazide [IF-1]. Yield, 86%, melting point, 256–258 °C, *δ*_H_ (600 MHz, DMSO) 12.20 (1H, d, *J* 17.6), 11.21 (1H, s), 8.62 (1H, s), 8.44 (1H, s), 7.93 (1H, d, *J* 8.1), 7.77 (1H, d, *J* 8.2), 7.51 (1H, t, *J* 7.4), 7.38–7.31 (2H, m), 7.25 (1H, s), 1.42 (4H, s), 1.28 (5H, s). ^13^C NMR (151 MHz, DMSO) *δ* 163.15, 154.81, 153.72, 151.92, 140.53, 135.92, 135.75, 130.60, 128.73, 128.35, 126.85, 125.96, 125.91, 123.92, 120.32, 116.99, 110.61, 40.07, 39.95, 39.82, 39.68, 39.54, 39.40, 39.26, 39.12, 34.71, 33.94, 31.33, 29.34. Elemental analysis calculated for C_26_H_30_N_2_O_3_ (418.23); C, 74.61; H, 7.23; N, 6.69; Found; C, 74.51; H, 7.37; N, 6.81.

#### 2.2.1 (*E*)-3-Hydroxy-*N*′-(4-nitrobenzylidene)-2-naphthohydrazide [IF-2]

Yield, 84%, melting point, 280–282 °C *δ*_H_ (600 MHz, DMSO) 12.17 (1H, s), 11.18 (1H, s), 8.55 (1H, s), 8.43 (1H, s), 8.31 (2H, d, *J* 8.3), 8.02 (2H, d, *J* 8.3), 7.92 (1H, d, *J* 8.1), 7.76 (1H, d, *J* 8.2), 7.51 (1H, t, *J* 7.4), 7.35 (2H, dd, *J* 16.1, 8.8). ^13^C NMR (125 MHz, DMSO) *δ* 163.83, 153.68, 147.99, 145.86, 140.55, 135.89, 130.67, 128.72, 128.32, 128.18, 126.86, 125.89, 124.15, 123.89, 120.83, 110.56. Elemental analysis calculated for C_18_H_13_N_3_O_4_ (335.09); C, 64.48; H, 3.91; N, 12.53; found; C, 64.62; H, 3.64; N, 12.41.

## 3. Results and discussion

### 3.1. Anion tracking study

This study comprises of analyzing the behavior of chemosensor toward a specific anion in the presence of group of anions. Here we have analyzed the behavior of IF-1 and IF-2 towards a range of anions in solution form such as F^−^, Cl^−^, Br^−^, CN^−^, SCN^−^, HSO_4_^−^, ClO_4_^−^, and AcO^−^, Both chemosensors showed color change from colorless to yellow for CN^−^ while IF-1 has shown color change for F^−^ and AcO^−^ ions as well hence former being more selective for CN^−^. This color change is most probably due to H-bonding between Schiff base and anion in case of F^−^ ion whereas CN^−^ interaction results due to H-bonding as well as deprotonation due to nucleophilic character of CN^−^ ions while selective response of IF-2 could be due to easy deprotonation at NH center that is further explained on the basis of UV data S.1. 7.†

### 3.2. UV-vis spectroscopic analysis

**Fig. 1:**
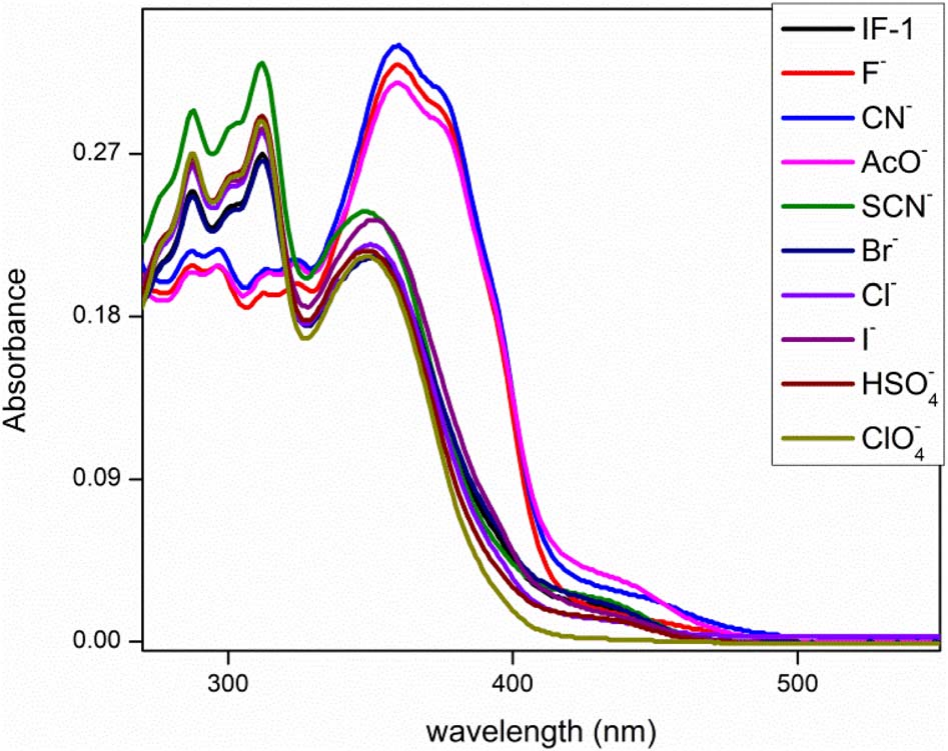
UV-vis selectivity of chemosensor IF-1 in CH_3_CN.

**Fig. 2:**
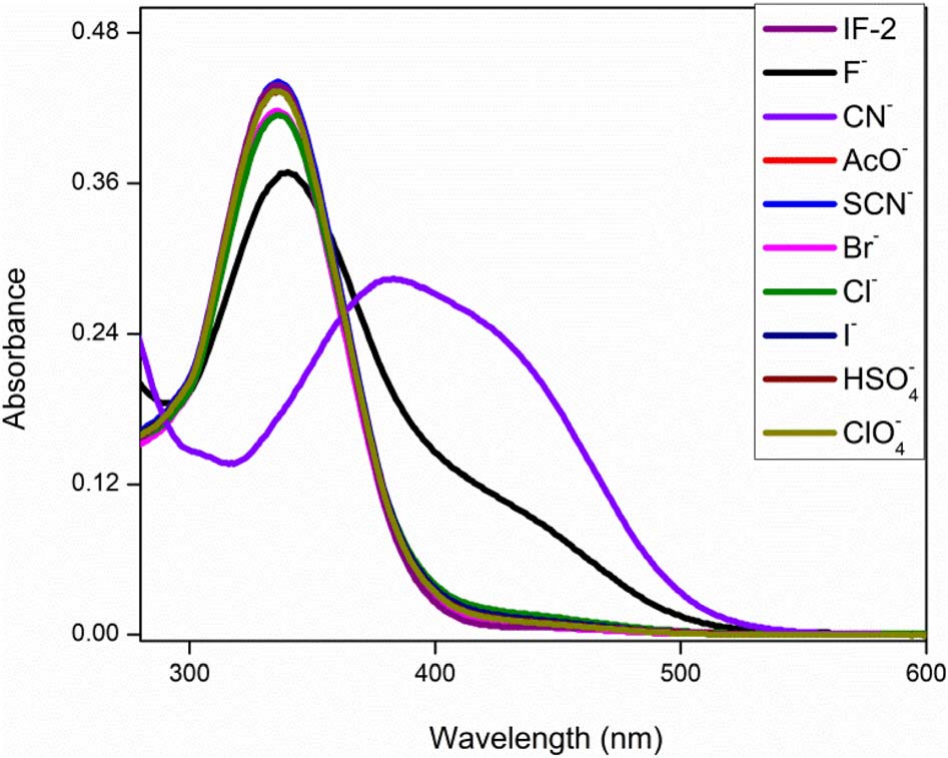
UV-vis selectivity of chemosensor IF-2 in CH_3_CN.

This analysis was done by taking solution of chemosensor in CH_3_CN. The chemosensor IF-2 displayed a poor selectivity for anions detection as illustrated in Fig. 1 while the chemosensor IF-2 displayed high selectivity toward CN^−^ ions as shown in Fig. 2. The chemosensor IF-2 displayed absorbance band at 335 nm. To study the chemo sensing behavior, CN^−^ was added into solution that shifted the *λ*_max_ towards higher value center at 420 nm. Presence of other anions didn't affect the absorption behavior of chemosensors IF-2. The color of the solution changed from colorless to yellow in 2 s on addition of CN^−^, ions hence this chemosensors have shown naked eye detection of CN^−^ ions in a very short time.

### 3.3. Competitive studies

**Fig. 3:**
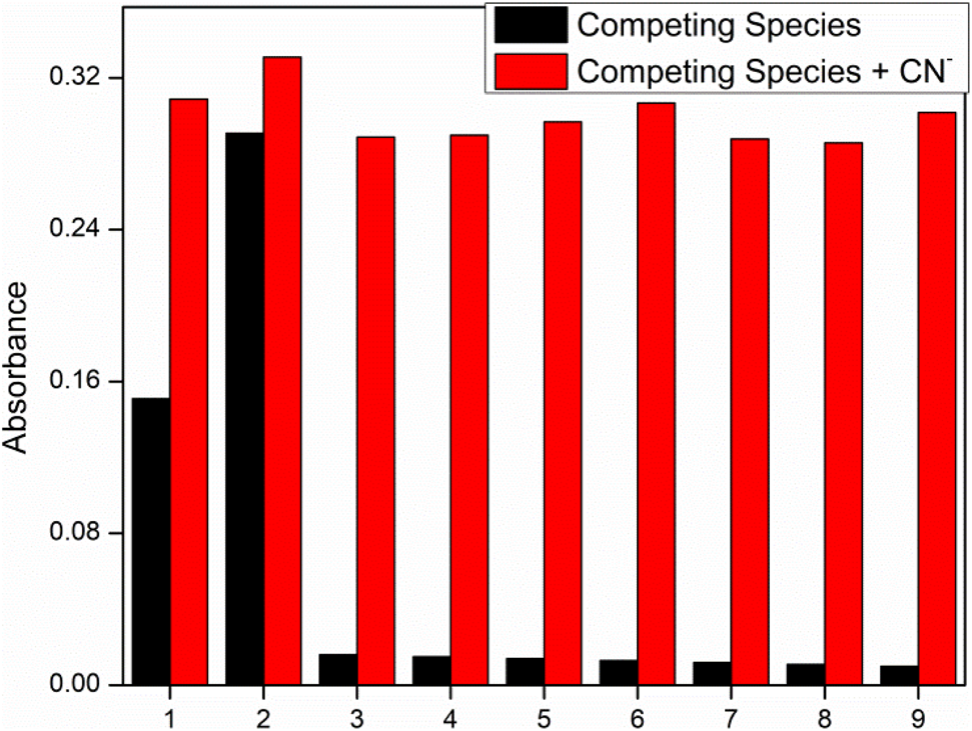
Competitive study of chemosensor IF-2 in CH_3_CN.

Competitive studies were used to study the selective behavior and sensing mechanism of our synthesized chemosensors. In this competitive experiment (Fig. 3) behavior of chemosensors was studied with 3 equivalent of CN^−^ ions in presence of other anions. Results has shown the excelling response of chemosensors IF-2 towards CN^−^ that was due to easy abstraction of hydrogen from NH and OH that was further confirmed by ^1^H NMR and titration studies. While other competing anions had a negligible effect on efficiency of our synthesized chemosensor.

### 3.4. UV-visible titration

**Fig. 4:**
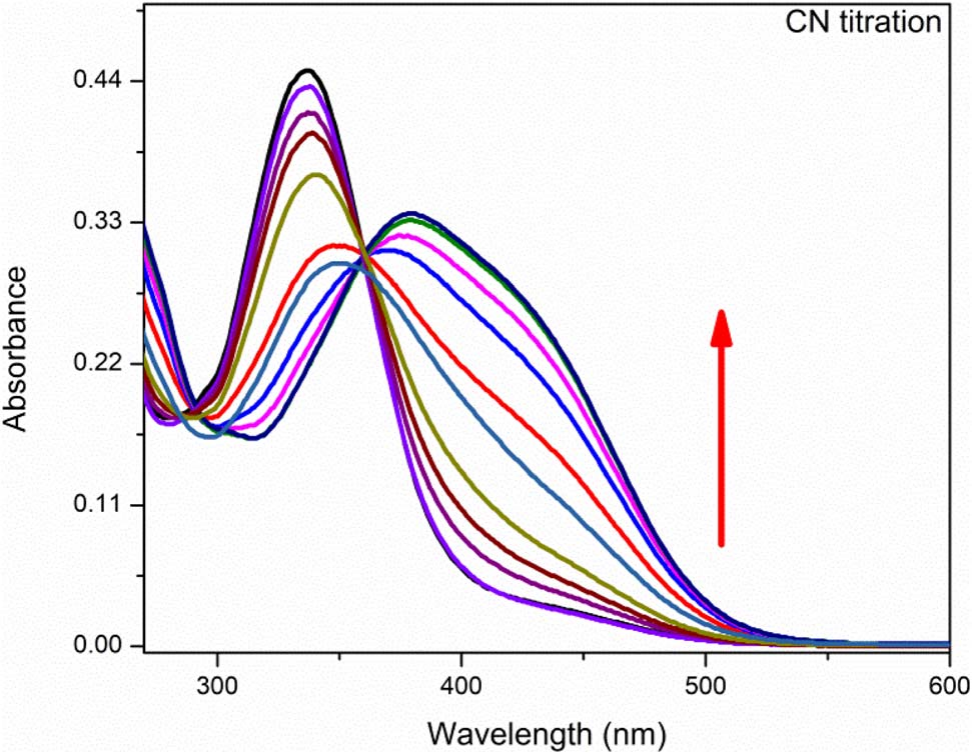
UV-vis titration of chemosensor IF-2 after the addition of 3 equiv. in CH_3_CN.

**Fig. 5:**
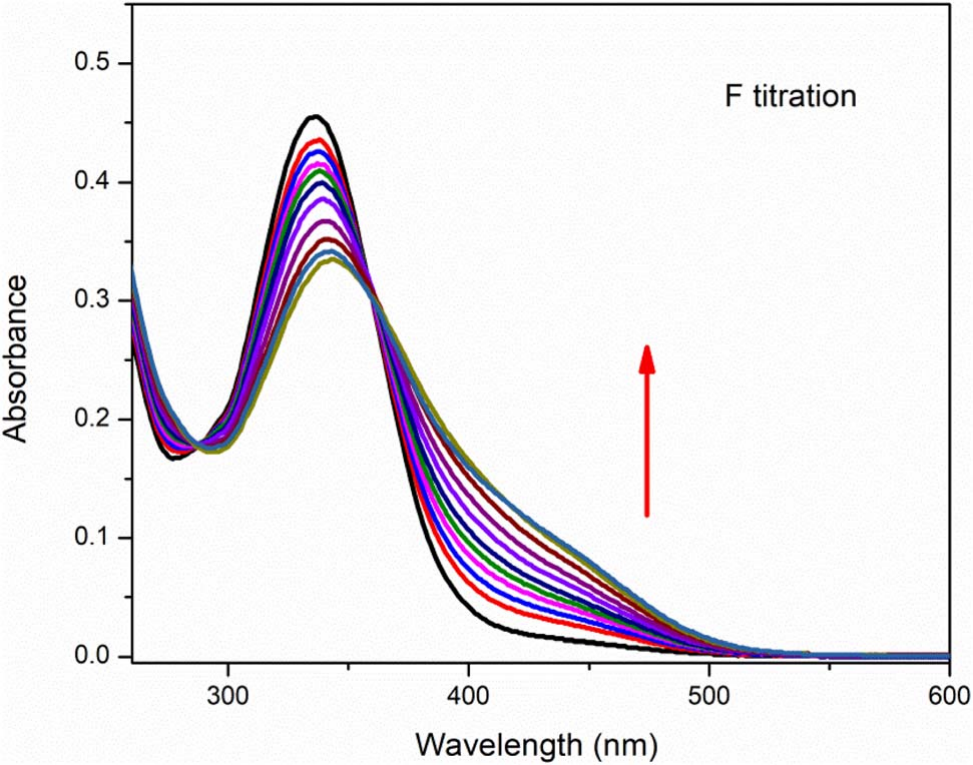
UV-vis titration of chemosensor IF-2 after the addition of 3 equiv. in CH_3_CN.

Anion binding ability of chemosensor was observed by UV-visible titration where acetonitrile was used as solvent (Fig. 4). In this titration the concentration of chemosensor was kept constant with gradual increase in concentration of CN^−^ ions from 0 to 3 equivalents. The graph showed a gradual decrease in absorption band centered at 335 nm (the absorption bands for chemosensors in absence of CN^−^ ions) while continuous increase in absorption band at 420 nm. The band around 420 nm was completely dominant when 3 eq. CN^−^ ions were added into solution. This gradual change in absorbance intensity showing a red shift describes the chemosensing activity of IF-2 towards CN^−^. The isosbestic point around 377 nm shows a stable complex formation. Similar titration was done with F^−^ ions as illustrated in (Fig. 5).

### 3.5. ^1^H-NMR titration

**Fig. 6:**
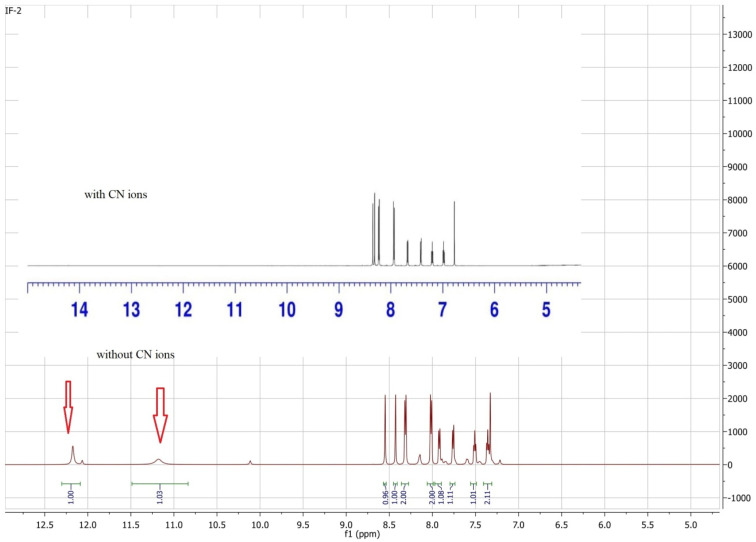
^1^H NMR spectra of IF-2 with and without addition of TBACN.

**Scheme 2:**
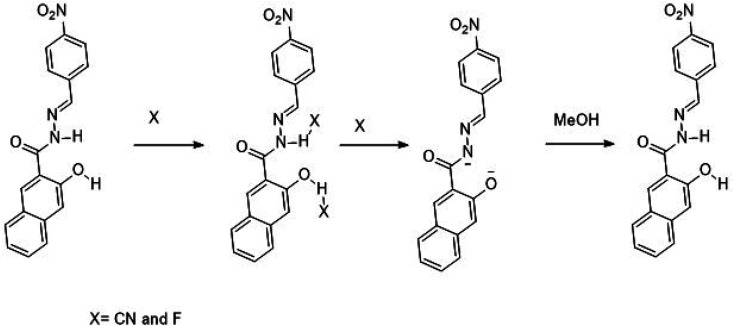
Proposed mechanism of sensing.

^1^H NMR titrations were performed to study the interaction of anion IF-2 that has clearly proved the deprotonation caused by CN^−^ ions. The ^1^H-NMR studies have shown a clear peak of N–H proton at 12.17 ppm and OH peak at 11.18 ppm for chemosensor IF-2 (Fig. 6). These peaks were completely disappeared when a solution of TBACN was added into it that confirms the deprotonation of N–H and OH. The shift in ^1^H NMR value for IF-2 describes the H-bonding formed between sensor and CN^−^ ion (Scheme 2).

### 3.6. Job's plot

**Fig. 7:**
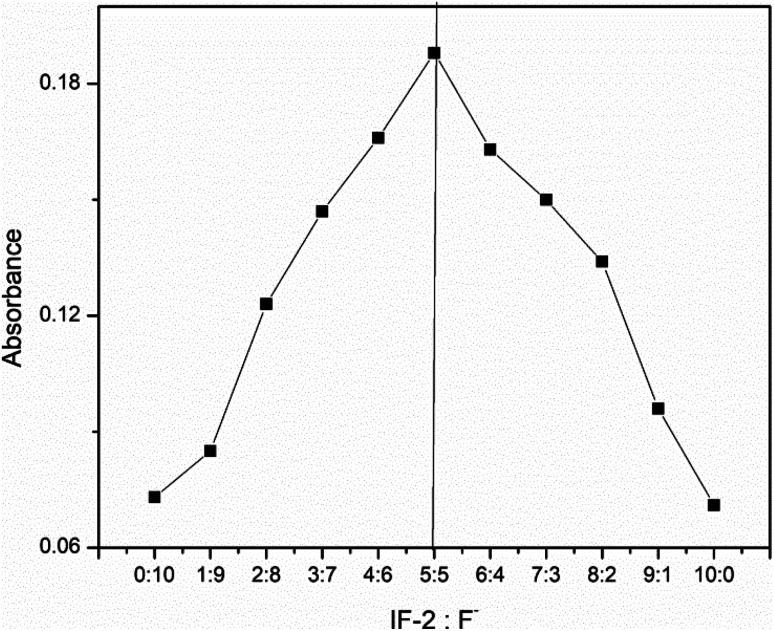
Job's plot of chemosensor IF-2.

Job's plot was used to calculate stoichiometric equation of newly synthesized chemosensor (IF-2) and CN^−^ ions. The stoichiometric ratios calculated for IF-2 were 1 : 1 (Fig. 7). Benesi–Hildebrand (B–H) was used to calculate the binding ratio of chemosensor and CN^−^ that is actually based on UV-vis titration. From here binding constants calculated for IF-2 are 4.77 × 10^4^ M^−1^. LOD (limit of detection) for CN^−^ was calculated using equation (LOD = 3*σ*/slope) that is measured as 8.2 μm for chemosensor IF-2 S.1-8-9.† The synthesis chemosensor have advantages over the previous reported probes as shown in Table 1.

**Table 1:** A comparison of limits of detection and binding constants of chemosensor with other reported sensors

References	Structure	Limit of detection (LOD)	Binding constant (*K*_a_) M^−^
33	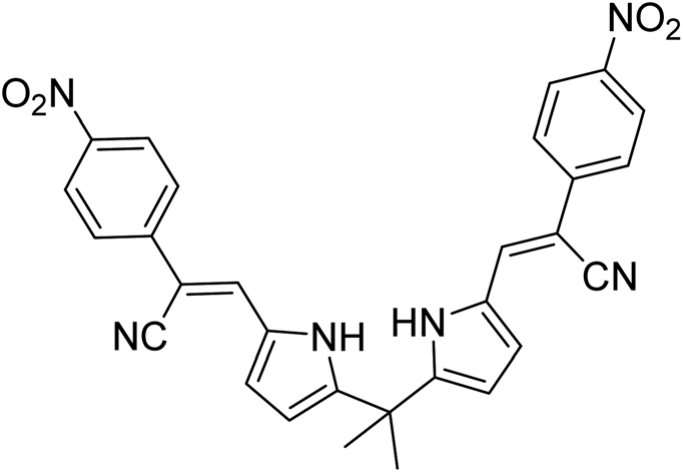	20.5 × 10^−6^	1.93 × 10^4^
34	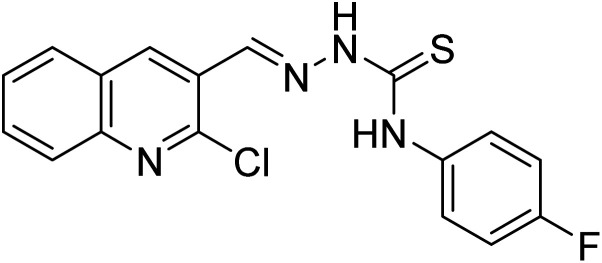	9.60 × 10^−6^	1.53 × 10^4^
35	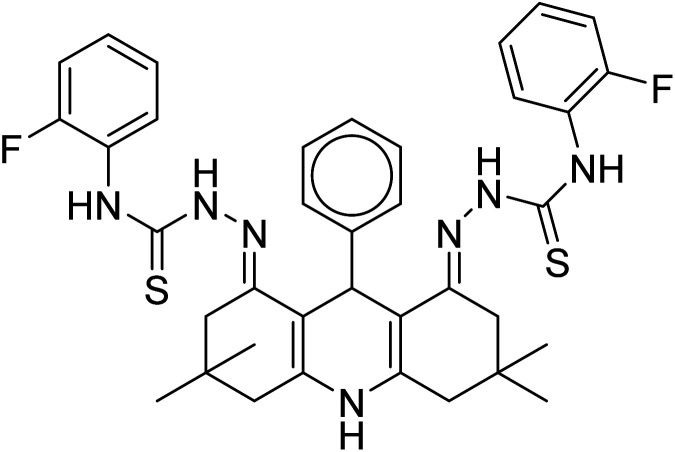	9.08 × 10^−5^	4.48 × 10^3^
36	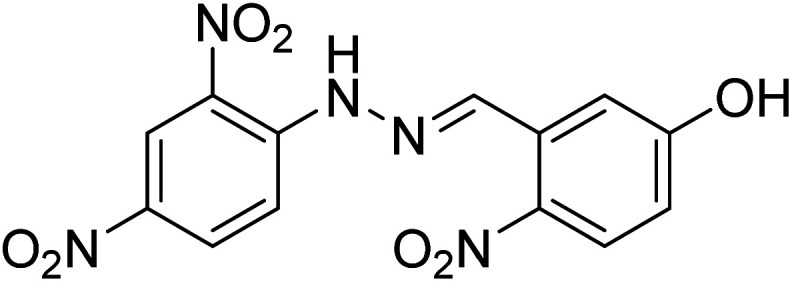	2.38 × 10^−5^	1.5 × 10^2^
37	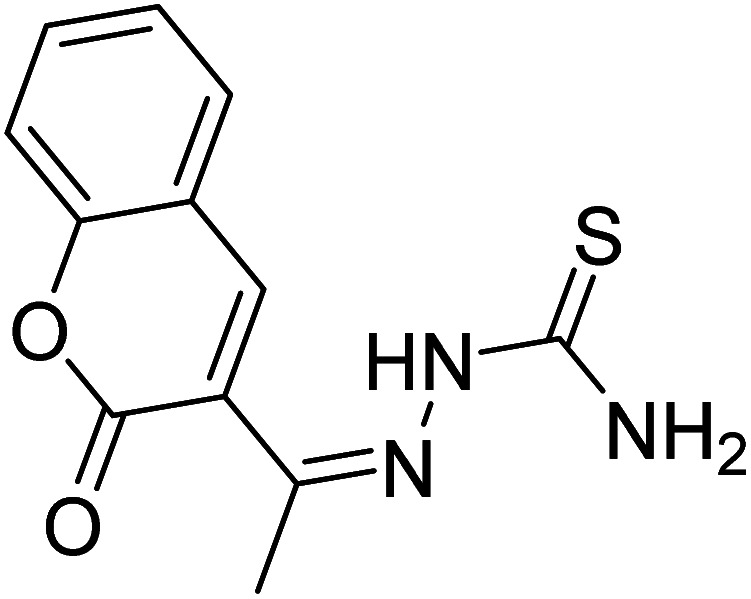	9.08 × 10^−6^	1.165 × 10^4^
38	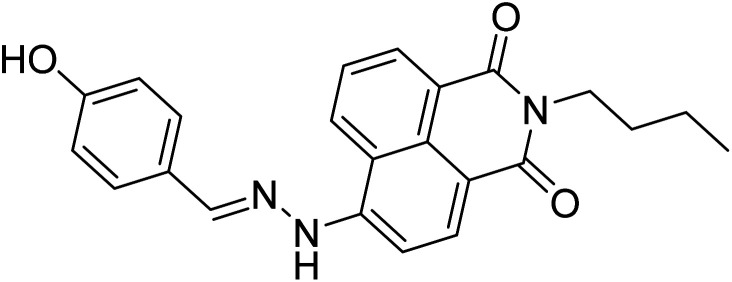	33.7 × 10^−6^	—
39	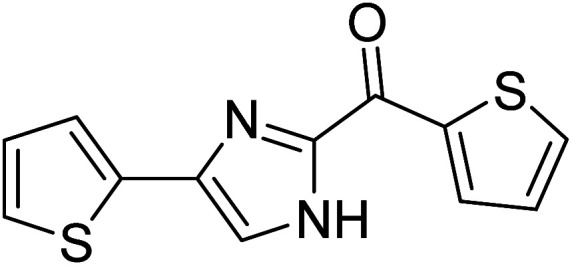	9.22 × 10^−6^	—
40	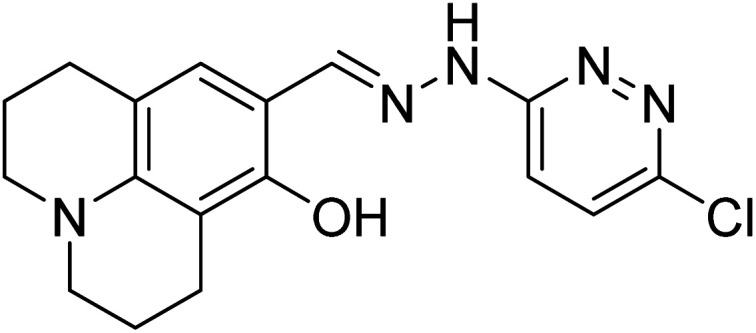	12.1 × 10^−6^	—
41	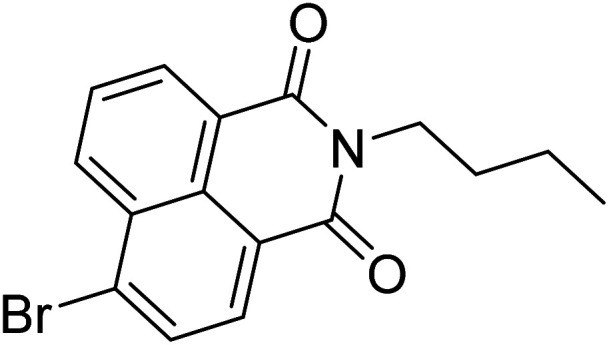	16.63 × 10^−6^	—
42	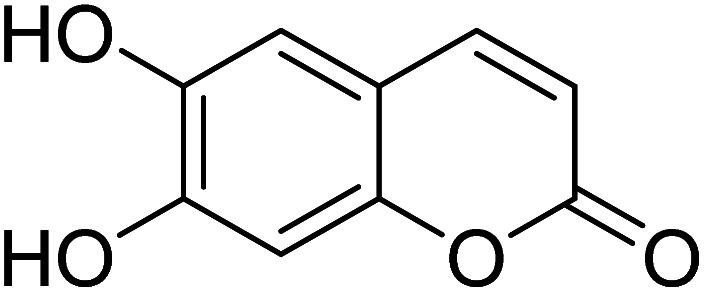	14.4	—
This work	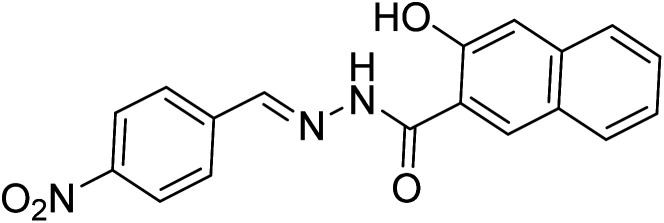	8.2 × 10^−6^	4.77 ×10^4^

### 3.7. Reverse reaction

**Fig. 8:**
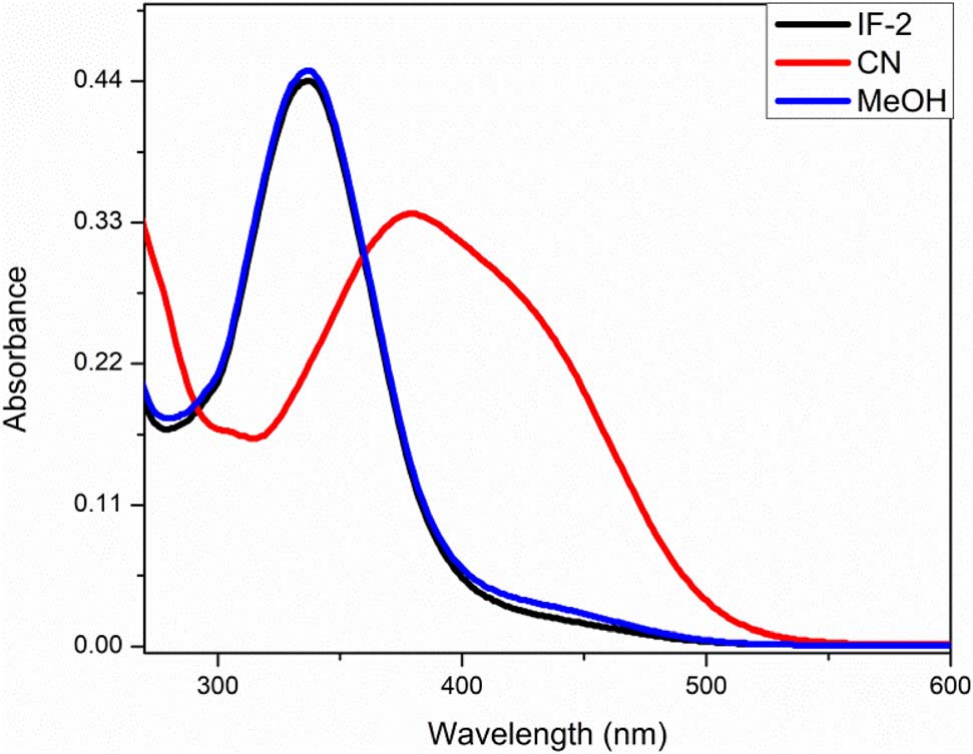
Reversible reaction of chemosensor IF-2 with MeOH.

Reversing the reaction can be described as regaining the original state of reaction that had gone through some change caused by CN^−^ ions. It is an interesting study that brings the reaction or chemosensor in their former state even when anions are present in the solution that is confirmed by UV studies. Reversibility of CN^−^ ions can be done using a strong proton donating agents such as in our studies we have used methanol as protonating agent. The former *λ*_max_ values of chemosensor IF-2 were appeared at 337 nm that were shifted on adding the CN^−^ to 420 nm. Now to check the reversibility of reaction 0.1 ml MeOH was added into solution of chemosensor and CN^−^ ions that started shifting the band towards its initial position showing a hyperchromic shift and restoring the original color of solution. The absorption bands appearing at 420 nm were completely disappeared now as shown in (Fig. 8). This absorption behavior shows that formerly depeotonated N–H are now provided with H donated by methanol regaining its original protonated state. Secondly it confirms that change in color of solution and absorption band was due to deprotonation caused by CN^−^.

### 3.8. Logic gate

**Fig. 9:**
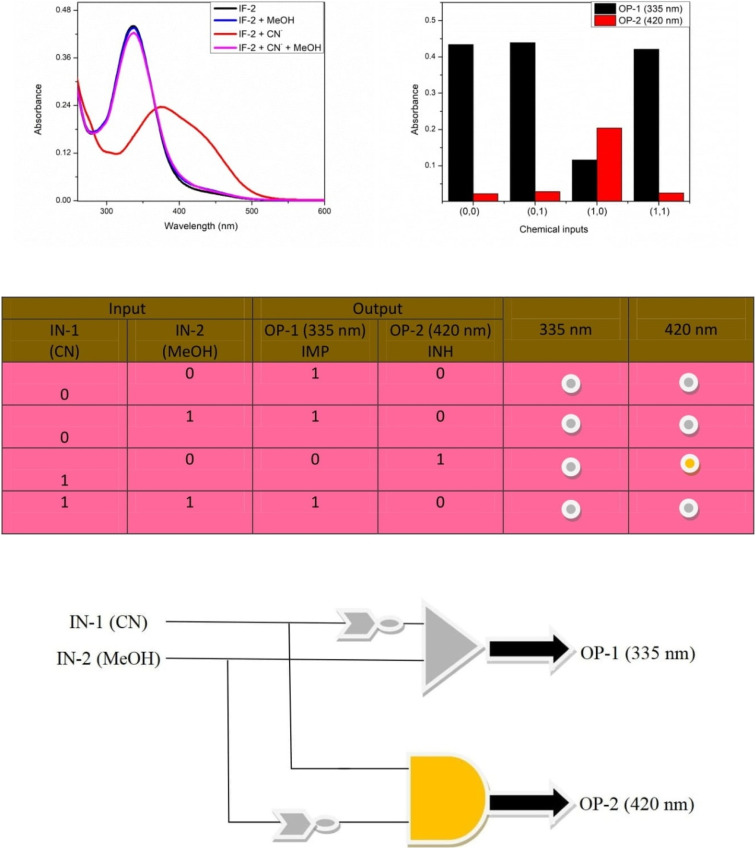
(A) Output signal of different input. (B) Absorbance outcome at different chemical inputs. (C) Truth table (D) Representation of IMP/INH logic circuit.

A combinatorial logic circuit with Implication (IMP) and Inhibition (INH) gates was created as a result of the chemosensor reversible reaction. IN-1 (F) and IN-2 (MeOH) were listed as the chemical inputs. The states of the chemosensor are represented by the numbers “0” and “1”. For chemosensor, the absorbance band at 335 nm was referred to as OP-1 and that at 420 nm as OP-2. The absence of absorbance at 420 when IN-1 and IN-2 are “0” (OP-2). This displays a status of OFF system. The absorbance at 420 nm remains unchanged when IN-1 and IN-2 are both “0”. This exhibits an OFF state as well. However, there is a shift in absorbance at 420 nm when IN-1 is “1” and IN-2 is “0,” which indicated an ON state. OP-2, an ON state, was shown by the sensor under this condition. When IN-1 and IN-2 are both “1,” the chemosensor once more showed a “OFF” status (OP-1). This is a 420 nm INH logic gate. A similar example using IMP logic at 335 nm was discovered. The absorbance at 335 nm was in the ON state when IN-1 and IN-2 were both “0.” And this also represents the ON state when IN-1 is “0” and IN-2 is “1.” (OP-1). However, there is a shift in absorbance at 335 nm, which represents the OFF state, when IN-1 is “1” and IN-2 is “0” (OP-1). Again, the chemosensor showed an ON state when IN-1 and IN-2 were both 1. (OP-1). The result of the IMP logic function simply completes INH logic (Fig. 9). These findings showed that chemosensor may find use in molecular devices.

### 3.9. Test strip

**Fig. 10:**
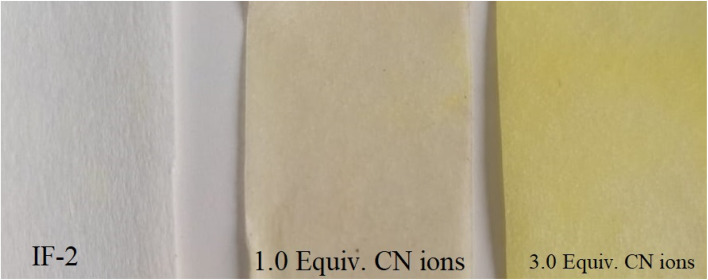
Test strip of chemosensor for CN^−^.

The production of test strips is one analytical use for recently synthesized chemosensor. These test strips may be used in labs or in the field to identify anions and analytes in a cost-effective manner. These strips were easily prepared using filter paper and sensing solution. A strip of filter paper was dipped in the chemosensor solution, dried, and then employed for anion detection, particularly for the CN^−^ ion. When strips were immersed in CN^−^ ion solution, their color changed from colorless to yellow (as seen in Fig. 10). These strips can therefore be utilized as indicators for CN ion detection due to a noticeable change in color.

### 3.10. α-Glycosidase inhibition

The synthesized compounds IF-1 and IF-2 were also screened to check inhibitory activity against α-glycosidase. This assay was carried out at micro level using Acarbose as standard inhibitors having IC_50_ value of 873.34 ± 1.67 μM. After preliminary screening IF-1 and IF-2, showed significant urease inhibition with IC_50_ values in range 1.33 ± 0.02 and 12.26 ± 0.18 μM.

## 4. Frontier molecular orbitals (FMOs) analysis

**Fig. 11:**
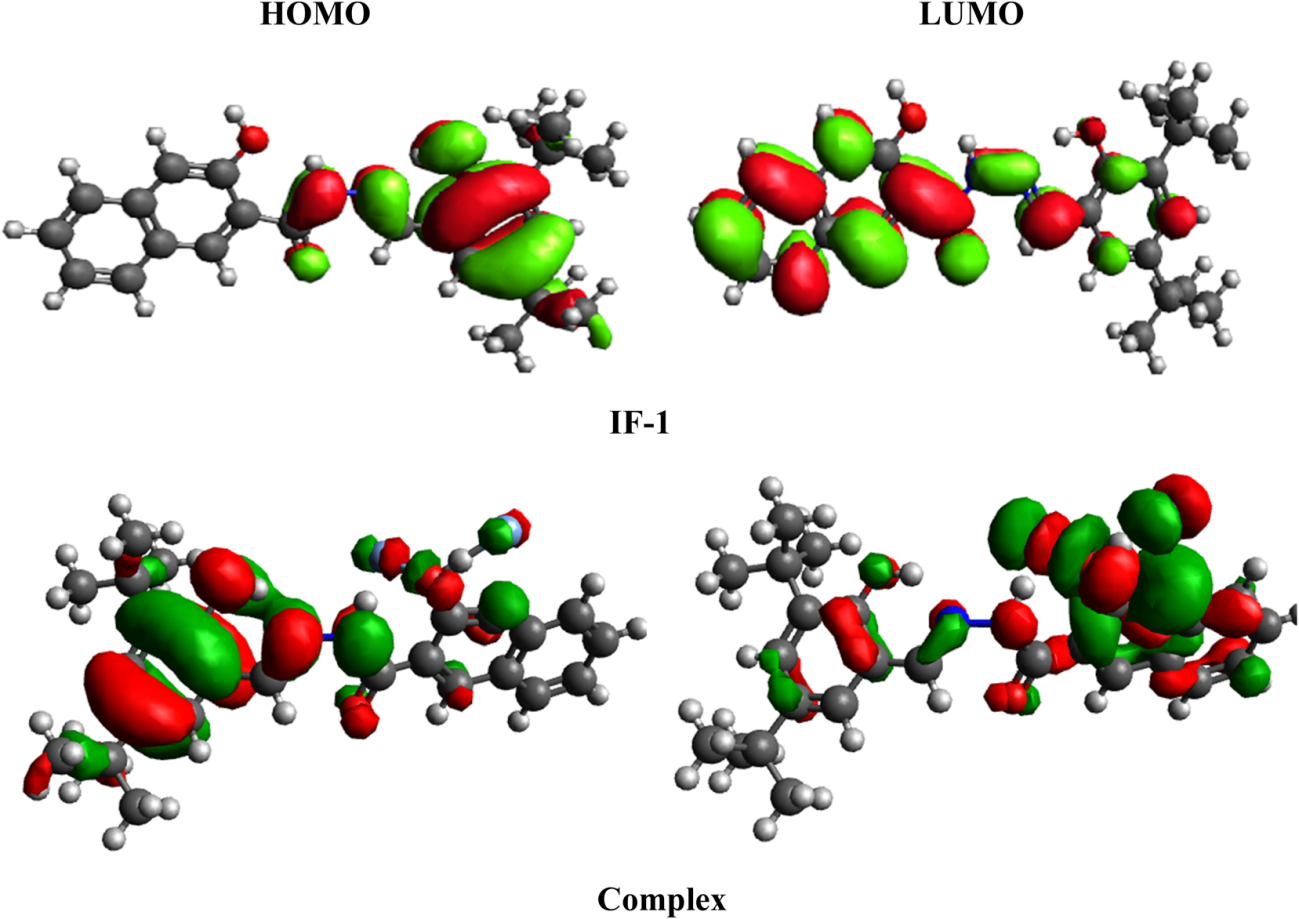
FMOs of IFF-1 sensor an it complex.

Frontier molecular orbitals (FMOs) analysis is a technique used to gain insights into the electron density distribution, kinetic stability and reactivity of sensors^43^ The HOMO, is typically associated with electron-donating capability, while the LUMO, is considered to have electron-accepting ability.^44^ The HOMO and LUMO band gap is critical parameter that influences the electronic behavior of molecules.^45^ The HOMO/LUMO band gap for IF-1 and its complex were found to be 4.1 and 0.776 eV, respectively, and same energy trend is study for higher molecular orbitals (Table S1†). Notably, the band gap of complex is observed to be smaller than that of IF-1, indicating a reduction in the band gap after the formation of interaction between sensor and fluoride ions. From the FMOs surface, a significant charge transfer is examined as show in Fig. 11. For HOMO charge is located over 3-hydroxy-2-naphthamide compound with 2,4-di-*tert*-butyl-6-methylphenol (1 : 1) the part while for LUMO this charge is significantly moves towards 2,4-di-*tert*-butyl-6-methylphenol in IF-1. While in complex, for HOMO the charge is located over the 3-hydroxy-2-naphthamide and significantly moves towards 2,4-di-*tert*-butyl-6-methylphenol as fluoride ion developed interaction with carbonyl group (Fig. 5). Same phenomena of charge transference is study for higher molecular orbitals (HOMO+1/LUMO−1 and HOMO+2/LUMO−2) as illustrated in Fig. S11†

### 4.1. Global reactivity parameters (GRPs) and molecular electrostatic potential (MEP) investigations

Global reactivity parameters (GRPs), such as the electronegativity (*X*),^46^ ionization potential (IP),^47^ electron affinity (EA),^48^ global softness (*σ*),^49^ hardness (*η*),^50^ chemical potential (*μ*),^51^ and electrophilicity index (*ω*),^52^ are valuable tools for evaluating the selectivity, stability, and reactivity of molecules.^53^ Results of Table S2† reveal that the global hardness value is 0.388 eV for complex, which is the lowest as compared to IF-1 (2.05 eV). This might be because of the development of interactions of fluoride ion with sensor, which make it kinetically least stable, more reactive and soft compound with low energy gap as compared to IF-1. Additionally, all the remaining parameter except global hardness were observed to be high for complex compound chromophore as compared to neutral, suggests increased reactivity and polarizability and development of interaction with ion.

**Fig. 12:**
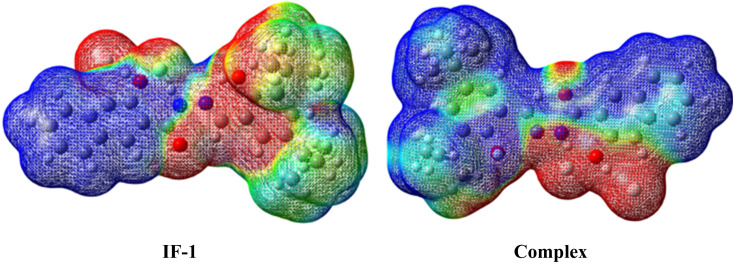
Molecular electrostatic potential (MEP) illustrations of IF-1 sensor and its complex.

The molecular electrostatic potential (MEP) is a 3D visualization tool for understanding charge distribution and non-covalent interactions^54,55^ MEP surface maps depict electron density, aiding in identifying surface reactivity and active sites for electrophilic and nucleophilic attacks through color-coded gradients. Colors range from dark red (negative potential) to blue (positive potential). The ascending order of magnitude of the electrostatic potential, from lowest to highest, is: blue < green < yellow < orange < red.^56^ Based on the MEP analysis, the nitrogen and oxygen atoms in both the neutral compound and complex compounds showed negative potentials, as indicated by red and yellow colors. This suggests that electrophilic attack is more likely in these regions due to the high electronegativity of these atoms. Furthermore, in the complex compound, the fluorine atom is also found to have a negative potential, indicating a development of interaction with hydrogen atom of carbonyl group. On the other hand, the blue color was mostly confined to the hydrogen and carbon atoms, indicating a maximum value of electrostatic potential. This makes them well-suited for nucleophilic attack. MEP surface maps depicted in (Fig. 12).

### 4.2. Quantum theory of atoms in molecules (QTAIM) analysis

**Fig. 13:**
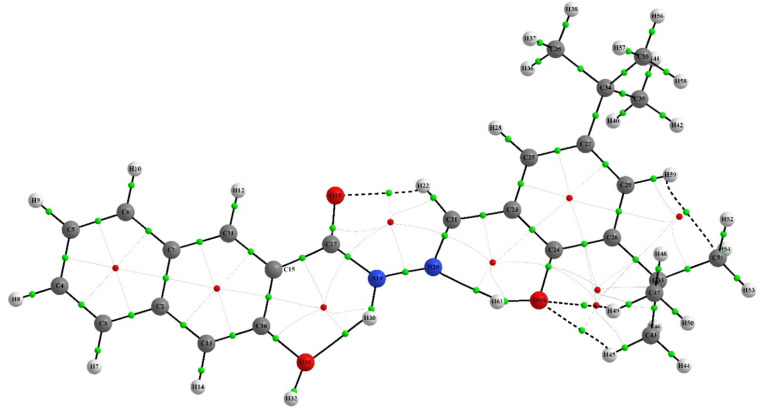
Schematic structure of AIM analysis of IF-1 sensor.

**Fig. 14:**
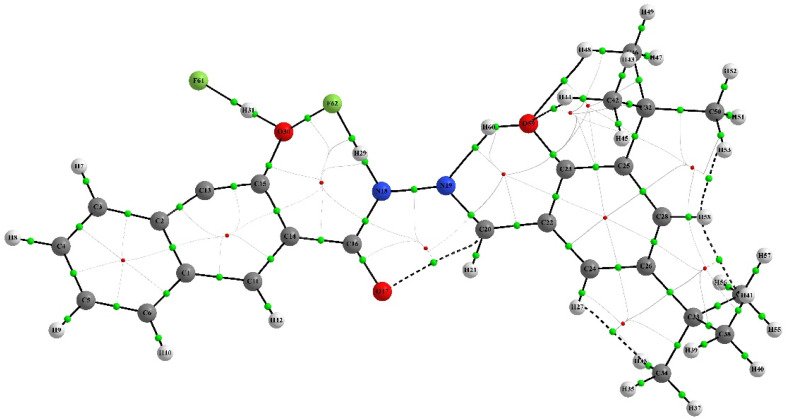
Schematic structure of AIM analysis of complex.

QTAIM analysis was employed to investigate the nature of the chemical bonding in the chemosensor and complex. The analysis focused on the electron density *ρ* at selected bond critical points (BCPs), which reveals the strength of chemical bonds.^57,58^ The Laplacian electron density ∇^2^ (*ρ*) and the ellipticity of the electron density *ε* were also studied to identify areas where the electronic charge is locally concentrated or depleted, and to provide a quantitative assessment of the anisotropy of the electron density, respectively. The QAIM analysis showed that intramolecular interactions, such as hydrogen bonds and non-covalent interactions, play a crucial role in stabilizing the structures of the IF-1 and complex compounds. This was supported by the presence of dashed bond paths (BPs) between the attractor atoms. As both structures shared similarities, the important interactions in both compounds were compared and analyzed. Tables 2 and 3 revealed that the intramolecular hydrogen bonds are the most significant interactions in both the neutral and complex structures. Further details can be found in ESI Tables S3 and S4.† The molecular graphs featuring the BCPs of selected bonds were presented in (Fig. 13 and 14).

**Table 2:** QAIM properties of the main intra- and intermolecular interactions for IF-1 Electronic density (*ρ*), Laplacian of density (∇^2^*ρ*), ellipticity (*ε*) and density of potential energy (*V*)

Bond	*ρ* (e/a^3^)	∇^2^*ρ* (e/a^5^)	*ε*	*V* (hartree e/a^3^)
O18–H22	+0.023161	+0.079227	+0.098176	−0.018668
H45–O60	+0.015346	+0.053709	+0.123776	−0.011619
H49–O60	+0.015196	+0.053293	+0.126335	−0.011490
C51–H59	+0.014580	+0.067393	+0.576660	−0.010382

**Table 3:** QAIM properties of the main intra- and intermolecular interactions for complex. Electronic density (*ρ*), Laplacian of density (∇^2^*ρ*), ellipticity (*ε*) and density of potential energy (*V*)

Bond	(e/a^3^)	∇^2^*ρ* (e/a^5^)	*ε*	*V* (hartree e/a^3^)
H41–H58	+0.013787	+0.056128	+0.600833	−0.008630
H53–H58	+0.017807	+0.076497	+0.786464	−0.012251
H27–H36	+0.016899	+0.074250	+0.759255	−0.011513
O17–C20	+0.014112	+0.055000	+2.565047	−0.010274
H44–O59	+0.009388	+0.036102	+0.346187	−0.006797

The QTAIM analysis of the compounds revealed differences in the electron density at the BCPs of their bonds. The O–H bonds in the neutral compounds (H45–O60, H8–H49, and O18–H22) exhibited higher electron density with *ρ* values of +0.023161, +0.015346, and +0.015196, respectively, compared to the complex compound (H44–O59) with a *ρ* value of +0.009388. This suggests stronger hydrogen bonding in the neutral compounds. In the complex compound, H–H bonding was observed at BCPs (H41–H58, H53–H58 and H27–H36) with relatively lower electron density compared to O–H bonds, indicating weaker interaction. Furthermore, C–H and C–O interactions were also observed in the neutral compounds (C51–H59 and O17–C20) with *ρ* values of +0.014580 and +0.014112, respectively, indicating the presence of multiple types of bonding in the compounds. It is noteworthy that although the O–H *ρ* values suggest weaker O–H bonds in the complex compound, there are still hydrogen bonding interactions present, as indicated by the positive *ρ* value for the H44–O59 bond. Hydrogen bonding is a special type of non-covalent interaction that plays an important role in many chemical processes, including biological systems.^59^ Therefore, the presence of hydrogen bonding interactions, even if they are weaker than in the neutral compounds, may still have important implications for the reactivity and properties of the complex compound. Overall, the QTAIM results provide valuable information about the nature of the chemical bonding in the neutral and complex compounds, highlighting the importance of intramolecular interactions and multiple types of bonding in these systems.

## 5. Conclusion

We have synthesized two novel chemosensors IF-1 and IF-2 for detection of CN^−^ in which IF-2 has clearly shown a very selective behavior towards CN^−^ ions. The synthesis process is single step condensation reaction between 3-hydroxy-2-naphthohydrazide and aldehyde derivatives giving a very good yield whose structure was confirmed using ^1^H NMR, IR, UV-vis spectroscopic techniques. The electron deficiency at neighboring aromatic ring in IF-2 makes the Schiff center more susceptible for deprotonation and hence more selective for CN^−^ that is further confirmed by the low detection limit (8.2 μM) and binding constant (4.77 × 10^4^ M^−1^) values. Job's plot confirmed the binding ratio as 1 : 1. Effective charge transference and development of non-covalent interactions were also study through DFT calculations. QTAIM findings confirmed the development of hydrogen bonding between sensors and its ions. Based on many tests and studies this chemosensor can be successfully used for detection of CN^−^ ion in real sample as well as manufacturing the detection tool *i.e.* test strips. This study will further pave the route for designing new and effective chemosensor for detection of CN^−^.

## Conflicts of interest

The authors declare no conflict of interest, financial or otherwise.

## Supplementary Material

RA-013-D3RA00788J-s001
