# Supplementary material for: Synthesis of novel 3-hydroxy-2-naphthoic hydrazones as selective chemosensors for cyanide ions
Source: RSC Adv. 2023 May 18;13(22):15208–21. doi: 10.1039/d3ra00788j (PMC10193203; doi:10.1039/d3ra00788j)
Supplement: RA-013-D3RA00788J-s001 [file RA-013-D3RA00788J-s001.pdf]

Electronic Supplementary Material (ESI) for RSC Advances.
This journal is © The Royal Society of Chemistry 2023

Supplementary Information for

Synthesis of Novel 3-Hydroxy-2-naphthoic hydrazones as Selective Chemosensors for Cyanide Ion

Rima D. Alharthy,[a] Ifra Urooj[b], Mussarat Tasleem[b], Muhammad Khalid*,[c,d] Muhammad Adnan Asghar,[e] Shaista Ijaz Khan[b], Muhammad Ajmal[b], Nadeem Ahmed[b] and Zahid Shafiq[b,*]

[a] *Department of Chemistry, Science & Arts College, Rabigh Branch, King Abdulaziz University,*
Rabigh 21911, Saudi Arabia

[b] *Institute of Chemical Sciences, Bahauddin Zakariya University, 60800 Multan, Pakistan*

[c]*Institute of Chemistry, Khwaja Fareed University of Engineering & Information Technology, Rahim Yar Khan, 64200, Pakistan*

[d]*Centre for Theoretical and Computational Research, Khwaja Fareed University of Engineering & Information Technology, Rahim Yar Khan, 64200, Pakistan*

[e]*Department of Chemistry, Division of Science and Technology, University of Education Lahore, Pakistan*

Content

1. ^{1}H NMR spectra of IF-1 ………..**S.I-1**
2. ^{13}C NMR spectra of IF-1………...,,,,,,,,,,,,,,,,...**S.1-2**
3. ^{1}H NMR spectra of IF-2 ………..**S.I-3**
4. ^{13}C NMR spectra of IF-2………..**S.1-4**
5. HRMS Spectra of IF-1…………………………………………………..……**S.1-5**
6. HRMS Spectra of IF-2……………………………………………….. **…...S.1-6**
7. Color change of IF-2………………………………………………….....**S.I.-7**
8. BH plot of chemosensor IF-2……………………………………….....……**S.I-8**
9. Linear plot of chemosensor IF-2……………………………………… **S.I-9**
10. HRMS Spectra of IF-2 after the addition of CN ion……………**S.I-10**
11. FMOs surfaces of IF-1 and its complex……………………**S.1-11**

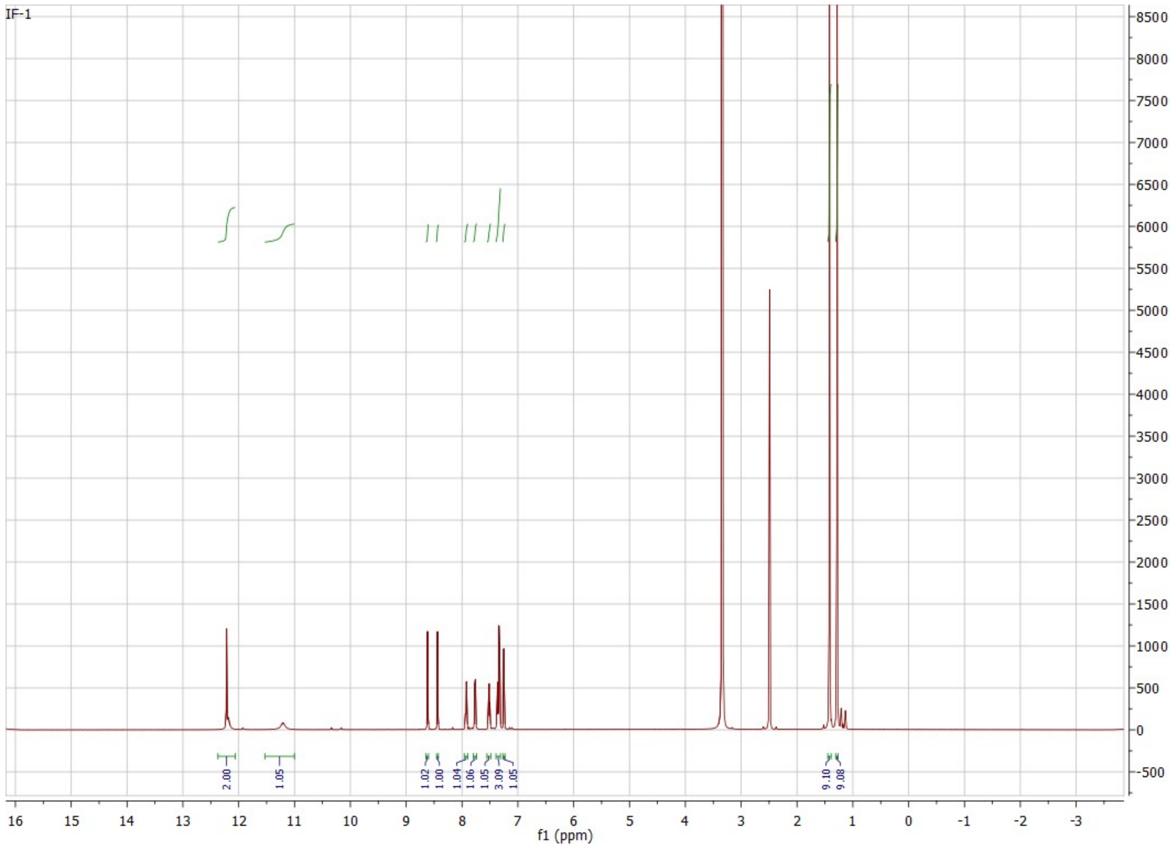

S.I-1 ^{1}H NMR spectra of IF-1

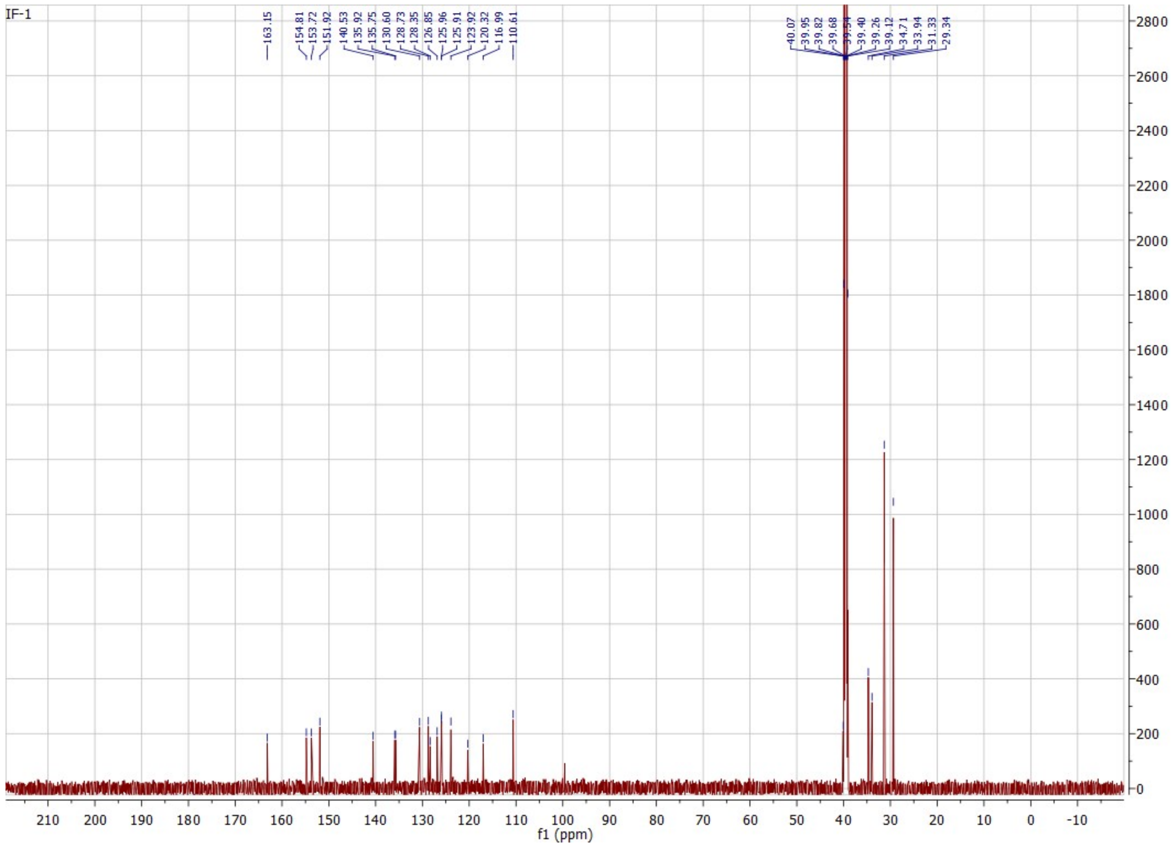

S.1-2 ^{13}C NMR spectra of IF-1

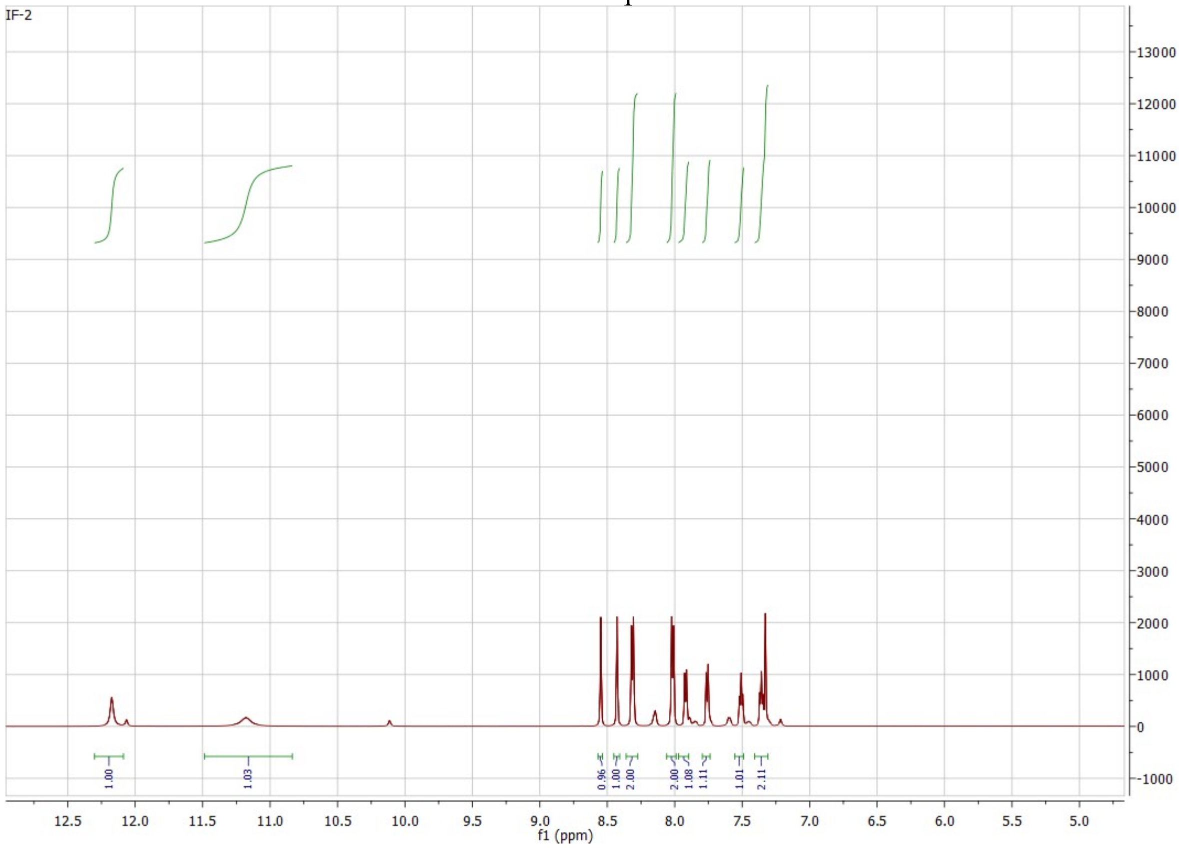

S.I-3 ^{1}H NMR spectra of IF-2

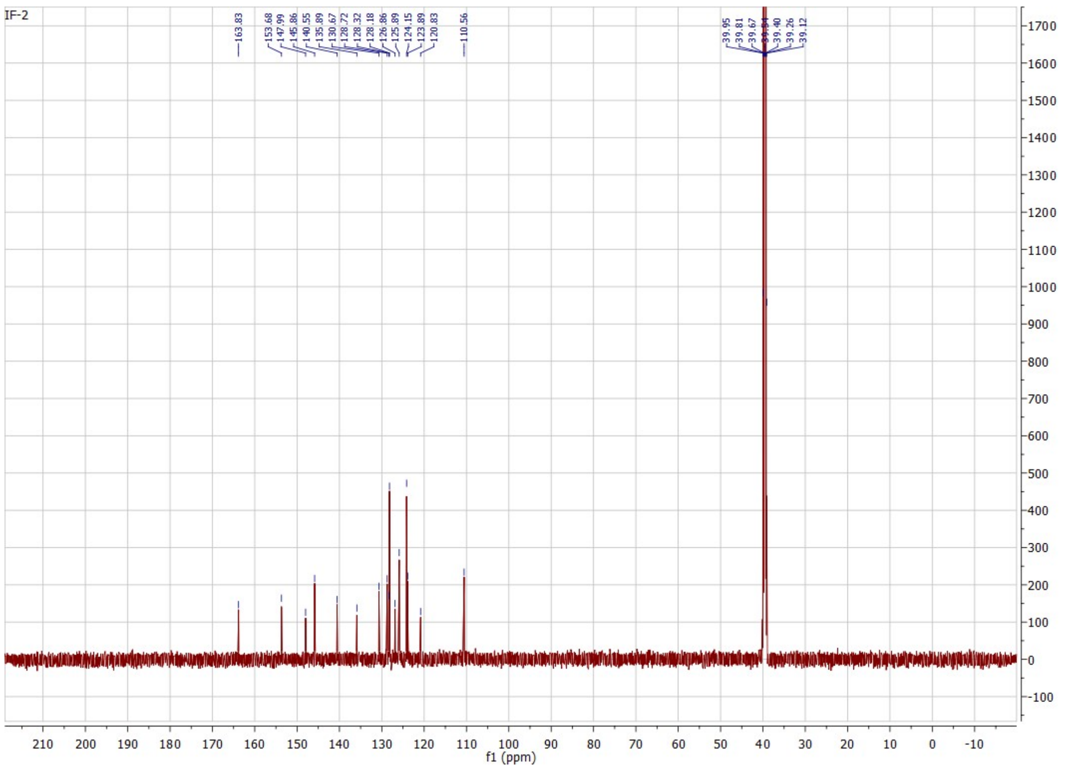

S.1-4 ^{13}C NMR spectra of IF-2

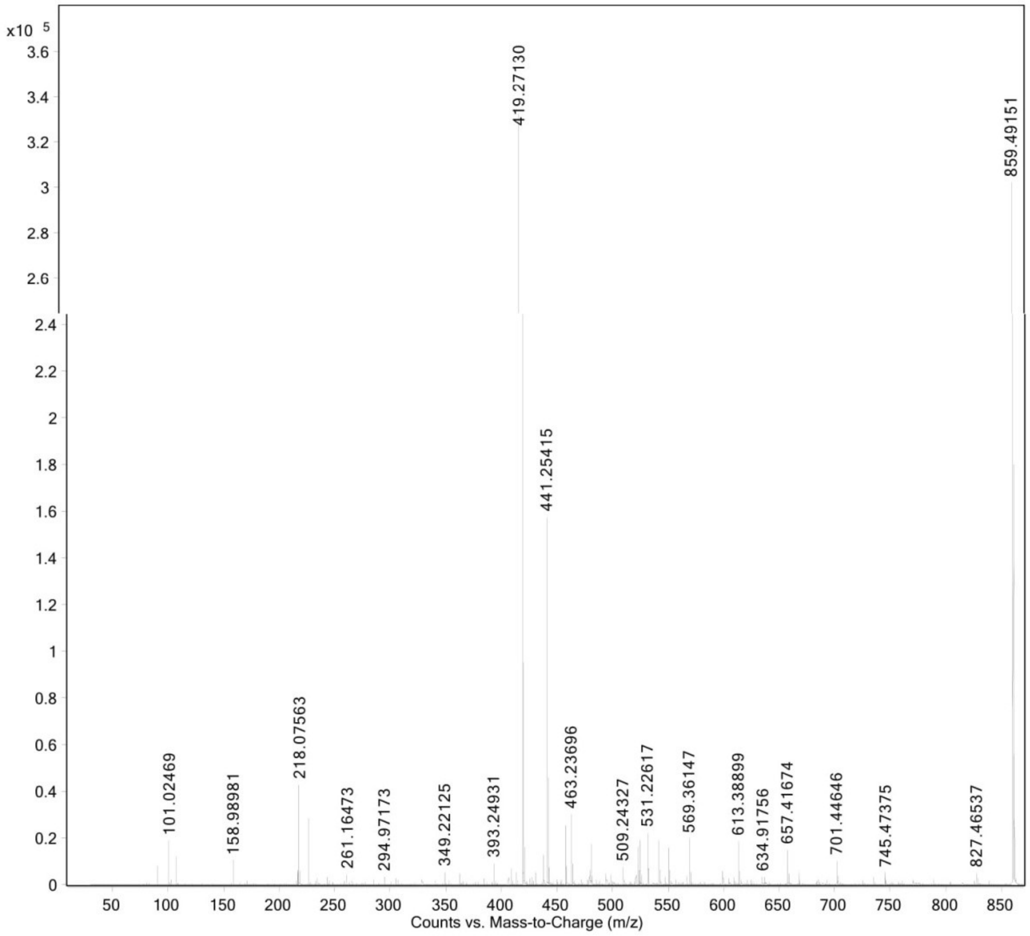

S.I-5 HRMS of IF-1

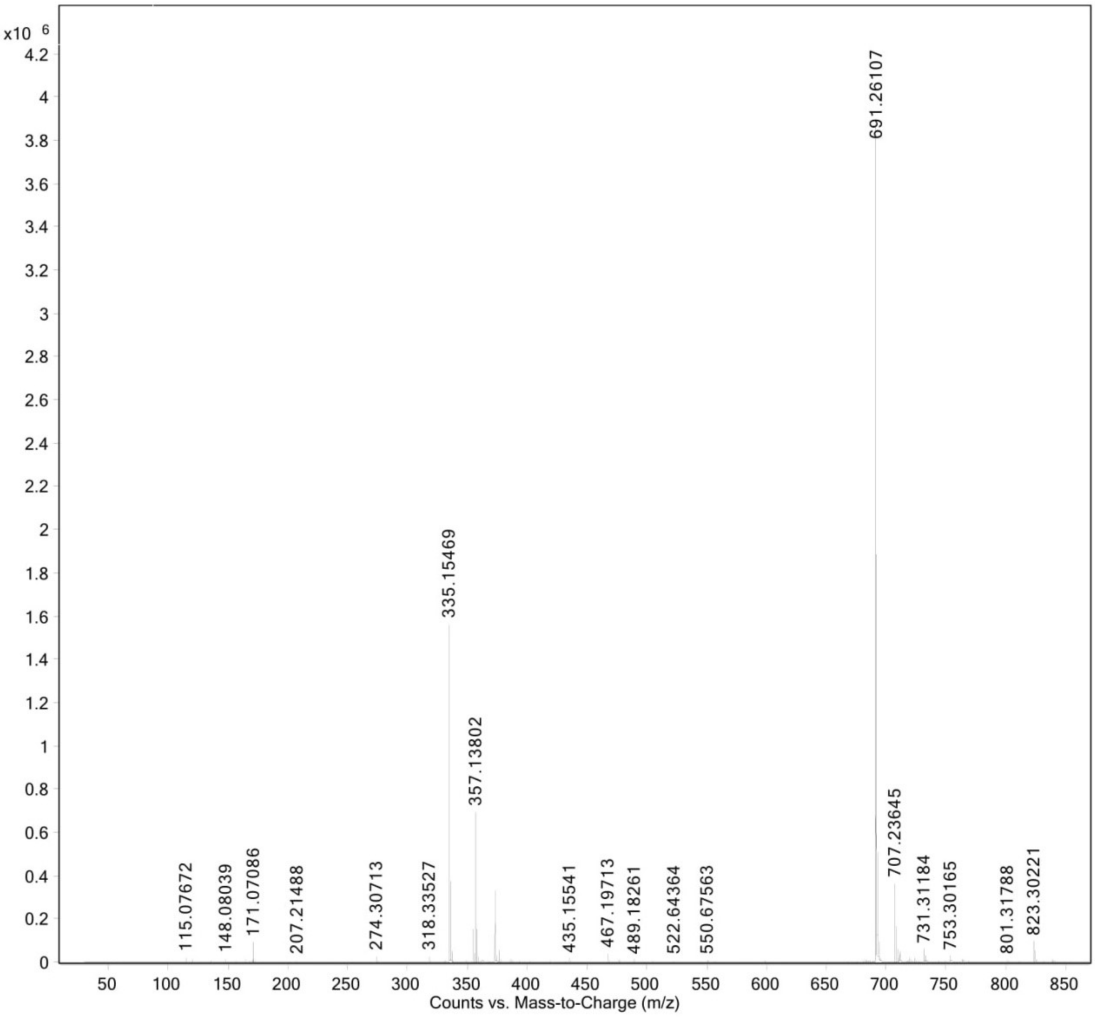

S.I-6 HRMS of IF-2

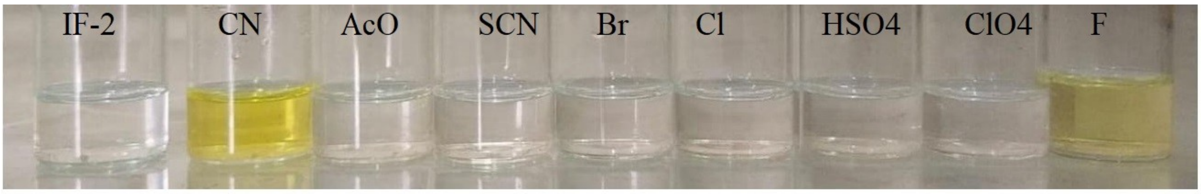

S.I.7 Color change of IF-2

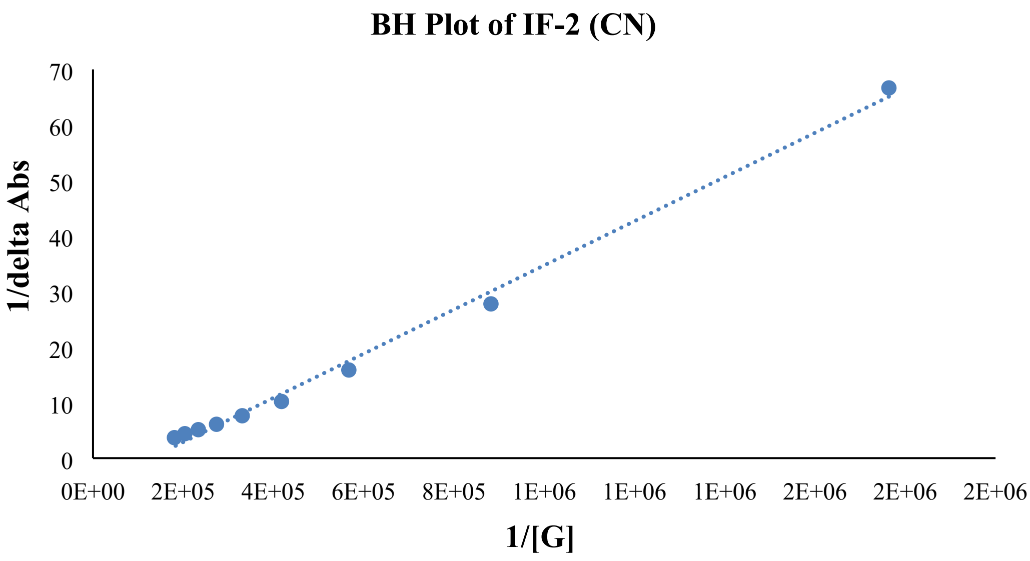

S.I-8 BH plot of chemosensor IF-2

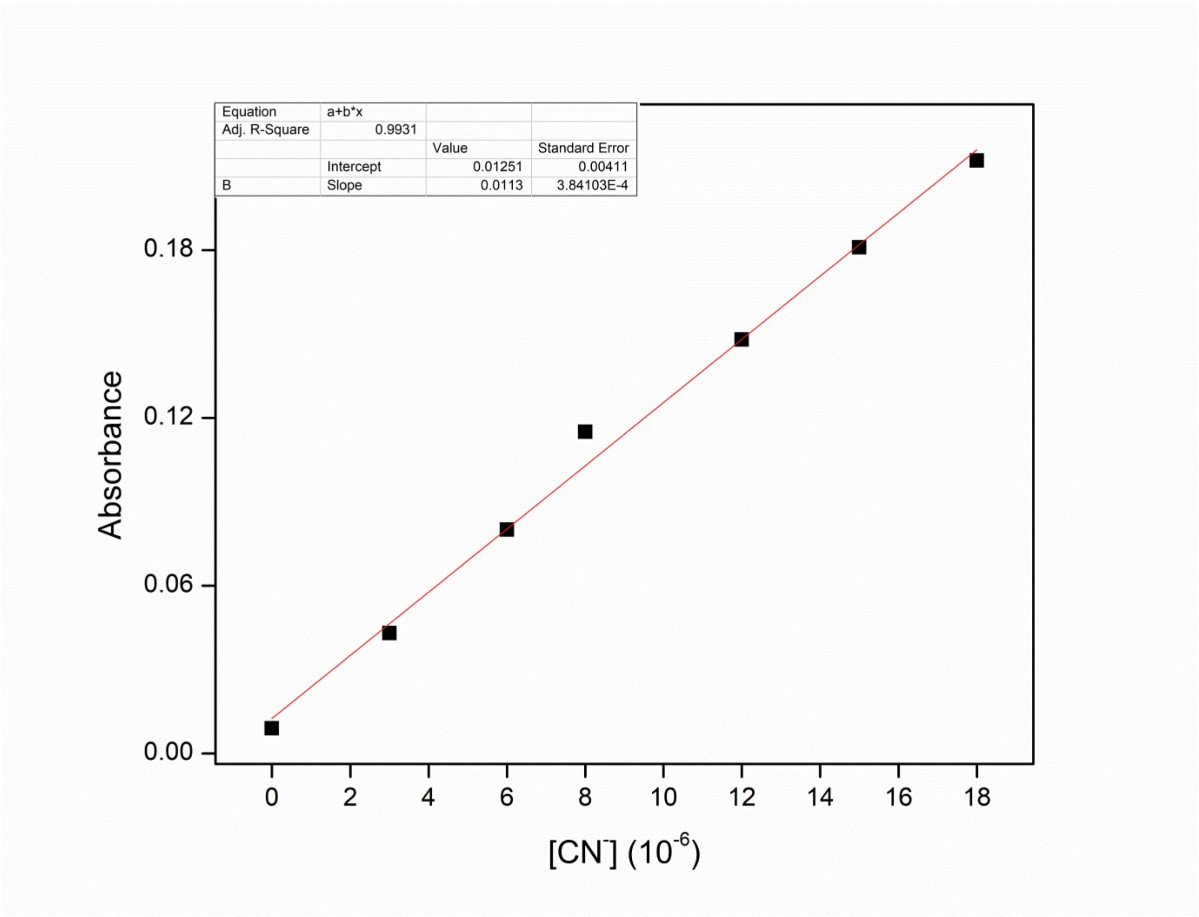

S.I-9 Linear plot of chemosensor IF-2

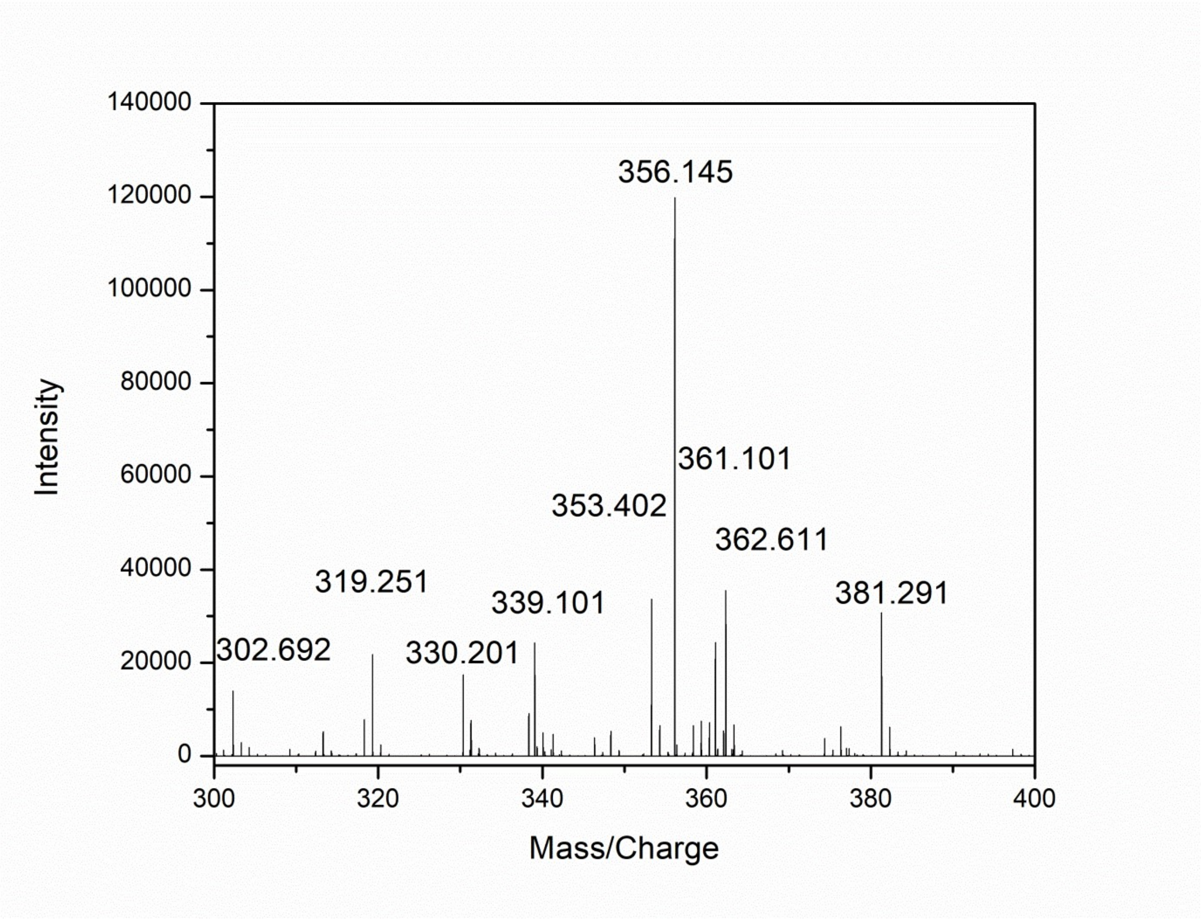

S.I-10 HRMS Spectra of IF-2 after the addition of CN^- ions

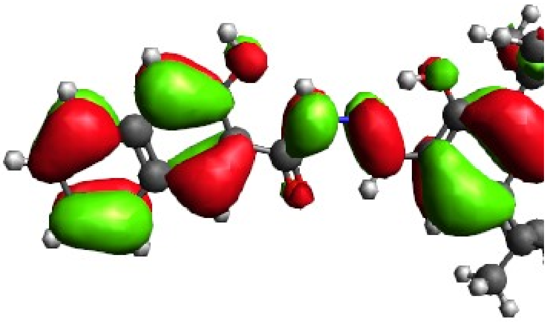

HOMO -1

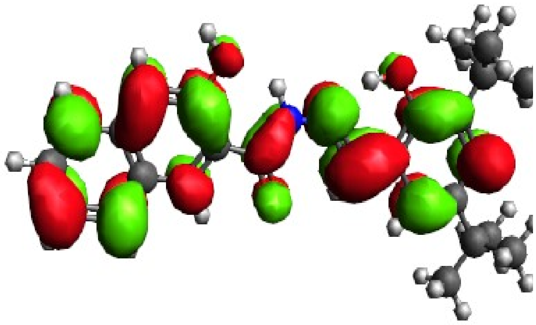

LUMO +1

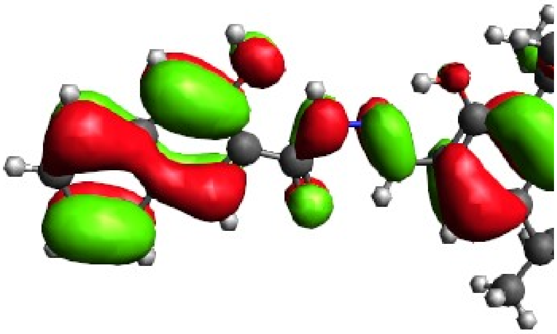

HOMO -2

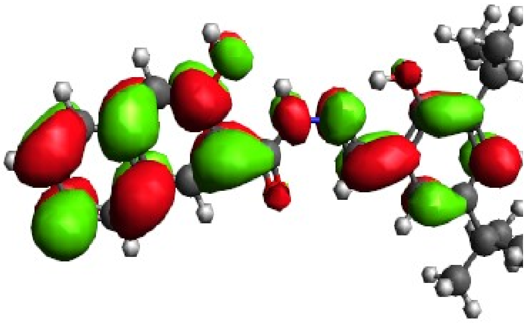

LUMO +2

Neutral

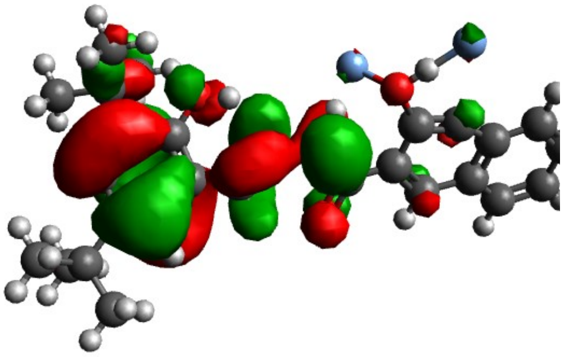

HOMO -1

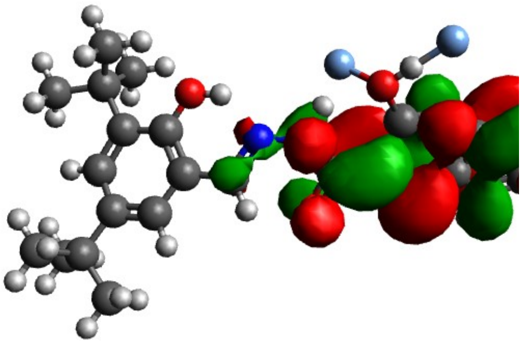

LUMO +1

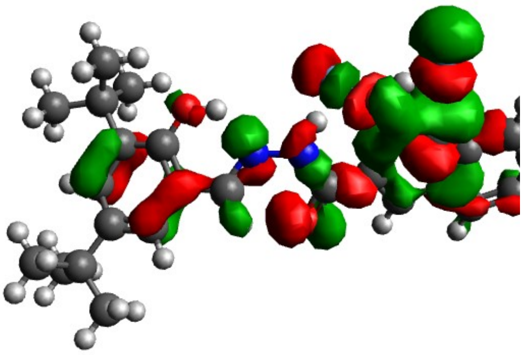

HOMO -2

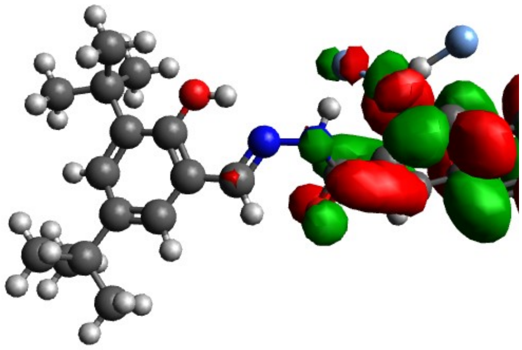

LUMO +2

Complex

Fig-S1-11: The HOMO-LUMO structures of IF-1 and it's Complex

Table S1: Calculated E_{HOMO}, E_{LUMO} and band gap of **IF-1** and its **Complex**

	**IF-1**		**Complex**	
**MOs**	***E*(*eV*)**	**Δ*E*(*eV*)**	***E*(*eV*)**	**Δ*E*(*eV*)**
HOMO	-5.717	4.1	-5.827	0.776
HOMO	-5.717	4.1	-5.827	0.776
LUMO+1	-0.855	5.479	-2.921	3.514
HOMO-1	-6.334		-6.435	
LUMO+2	-0.419	5.969	-1.867	4.944
HOMO-2	-6.388		-6.811	

Table S2: Global reactivity descriptors of IF-1 and its complex; unit in *eV*

**GRPs**	**IF-1**	**Complex**
IP	5.717	5.827
EA	1.617	5.051
X	3.667	5.439
η	2.05	0.388
μ	-3.667	-5.439
ω	3.279	38.122
σ	0.243	1.289

Table S3. AIM properties of the main intra- and intermolecular interactions for **test**. Electronic density (ρ), Laplacian of density ($\nabla^2\rho$), ellipticity (ε) and density of potential energy (V).

BCP	Bond	ρ (e/a³)	$\nabla^2\rho$ (e/a⁵)	ε	V (hartree.e/a³)
1	C15 - C17	+0.260591	-0.633727	+0.109318	-0.279848
2	C1 - C2	+0.298101	-0.783177	+0.145642	-0.370323
3	C3 - C4	+0.322605	-0.884760	+0.247868	-0.446372
4	C2 - C3	+0.300853	-0.797958	+0.151006	-0.378164
5	C4 - C5	+0.300411	-0.793371	+0.153115	-0.379662
6	C1 - C11	+0.304448	-0.816547	+0.143797	-0.386473
7	C5 - C6	+0.323276	-0.888397	+0.247176	-0.448326
8	C1 - C6	+0.300200	-0.795053	+0.149703	-0.375710
9	C2 - C13	+0.300761	-0.793071	+0.167232	-0.380027
10	C3 - H7	+0.278226	-0.947581	+0.016918	-0.325957
11	C4 - H8	+0.280494	-0.967125	+0.008441	-0.328379
12	C11 - C15	+0.319176	-0.863023	+0.230587	-0.436137
13	C5 - H9	+0.280229	-0.963506	+0.014491	-0.328763
14	C17 - O18	+0.400304	+0.010220	+0.084542	-1.365782
15	C6 - H10	+0.279350	-0.960453	+0.010612	-0.326233
16	C17 - N19	+0.315764	-0.973627	+0.132504	-0.718714
17	C11 - H12	+0.282540	-1.006134	+0.004621	-0.329534
18	C15 - C16	+0.296031	-0.782293	+0.198340	-0.369310
19	C13 - C16	+0.324880	-0.907016	+0.320180	-0.460855
20	C13 - H14	+0.273586	-0.907779	+0.029835	-0.321925
21	C16 - O31	+0.280437	-0.229374	+0.025085	-0.788371
22	N19 - N20	+0.345595	-0.617600	+0.071904	-0.507204
23	N20 - C21	+0.358345	-0.572297	+0.190753	-1.065192
24	O18 - H22	+0.023161	+0.079227	+0.098176	-0.018668
25	C21 - H22	+0.286344	-1.053367	+0.013619	-0.335000
26	C21 - C23	+0.282920	-0.730376	+0.119934	-0.331092
27	C23 - C25	+0.304954	-0.804367	+0.191732	-0.393287
28	C24 - O60	+0.298358	-0.271880	+0.002879	-0.856314
29	C23 - C24	+0.305390	-0.816512	+0.235346	-0.392414
30	C25 - H28	+0.279414	-0.957926	+0.012279	-0.328965
31	C25 - C27	+0.316586	-0.850063	+0.231697	-0.429727
32	C27 - C29	+0.304925	-0.801158	+0.180219	-0.394769
33	C26 - C33	+0.245742	-0.562991	+0.031725	-0.252964
34	C24 - C26	+0.305669	-0.825299	+0.225395	-0.394250
35	C29 - H59	+0.281265	-0.971260	+0.008712	-0.333769
36	C27 - C34	+0.249085	-0.578434	+0.031556	-0.257286
37	C34 - C35	+0.248467	-0.573394	+0.001469	-0.257241
38	C26 - C29	+0.311200	-0.818976	+0.224720	-0.415608
39	C51 - H59	+0.014580	+0.067393	+0.576660	-0.010382
40	N19 - H30	+0.343432	-1.853141	+0.052946	-0.563466
41	H30 - O31	+0.030738	+0.109129	+0.064231	-0.025906
42	O31 - H32	+0.361821	-2.079663	+0.021704	-0.670397
43	C33 - C51	+0.247796	-0.569810	+0.000729	-0.256127
44	C35 - H36	+0.271446	-0.885890	+0.006845	-0.317395
45	C35 - H37	+0.271473	-0.886033	+0.006798	-0.317468
46	C35 - H38	+0.271638	-0.886691	+0.006027	-0.318116
47	C34 - C39	+0.245055	-0.557986	+0.002874	-0.252039
48	C39 - H40	+0.271818	-0.889965	+0.007724	-0.317241
49	C39 - H41	+0.270678	-0.879930	+0.007516	-0.316103

50	C39 - H42	+0.272135	-0.890573	+0.007487	-0.319007
51	C33 - C43	+0.243879	-0.552569	+0.006145	-0.250099
52	C43 - H44	+0.270070	-0.873727	+0.011126	-0.316024
53	H45 - O60	+0.015346	+0.053709	+0.123776	-0.011619
54	C43 - H45	+0.276594	-0.931694	+0.007932	-0.324465
55	C43 - H46	+0.270844	-0.879973	+0.011860	-0.316812
56	C33 - C47	+0.243895	-0.552648	+0.006142	-0.250131
57	C47 - H48	+0.270875	-0.880318	+0.011833	-0.316840
58	C47 - H50	+0.270110	-0.874007	+0.011081	-0.316122
59	H49 - O60	+0.015196	+0.053293	+0.126335	-0.011490
60	C47 - H49	+0.276623	-0.931848	+0.007937	-0.324561
61	C51 - H53	+0.271854	-0.888478	+0.005117	-0.318537
62	C51 - H52	+0.271190	-0.883421	+0.006940	-0.317259
63	C51 - H54	+0.271202	-0.883418	+0.006917	-0.317301
64	C34 - C55	+0.245081	-0.558026	+0.002913	-0.252097
65	C55 - H56	+0.270692	-0.880032	+0.007472	-0.316134
66	C55 - H57	+0.271845	-0.890253	+0.007708	-0.317272
67	C55 - H58	+0.272072	-0.890144	+0.007459	-0.318870
68	N20 - H61	+0.046131	+0.126499	+0.044858	-0.035598
69	O60 - H61	+0.335223	-1.951311	+0.017202	-0.637722

Table S4. AIM properties of the main intra- and intermolecular interactions for **mode**. Electronic density (ρ), Laplacian of density ($\nabla^2\rho$), ellipticity (ε) and density of potential energy (V).

**BCP**	**Bond**	ρ **(e/a^3)**	$\nabla^2\rho$ **(e/a^5)**	ε	V **(hartree.e/a^3)**
1	C1 - C2	+0.317371	-0.885953	+0.151275	-0.431628
2	C2 - C3	+0.302809	-0.818853	+0.137741	-0.389187
3	C4 - C5	+0.295493	-0.770641	+0.130857	-0.365201
4	C4 - H8	+0.281963	-0.986086	+0.006340	-0.328468
5	C3 - C4	+0.335519	-0.964180	+0.241055	-0.481468
6	C1 - C11	+0.253939	-0.561839	+0.152795	-0.274877
7	C5 - C6	+0.317012	-0.850813	+0.235586	-0.430991
8	C1 - C6	+0.302524	-0.809074	+0.143395	-0.384199
9	C2 - C13	+0.342954	-1.062420	+0.114489	-0.555937
10	C3 - H7	+0.291268	-1.066905	+0.013027	-0.350769
11	C5 - H9	+0.294536	-1.082264	+0.006201	-0.361119
12	C6 - H10	+0.295836	-1.092912	+0.010342	-0.367308
13	C11 - C14	+0.259835	-0.553294	+0.254361	-0.297032
14	C11 - H12	+0.292531	-1.096101	+0.020349	-0.354106
15	C14 - C15	+0.303607	-0.815316	+0.177834	-0.404719
16	C13 - C15	+0.387336	-1.291055	+0.257111	-0.690716
17	C14 - C16	+0.305752	-0.878997	+0.111766	-0.379380
18	C16 - O17	+0.363952	-0.373064	+0.090185	-1.127424
19	C16 - N18	+0.247157	-0.520242	+0.163607	-0.318680
20	N18 - N19	+0.294064	-0.433909	+0.062124	-0.396424
21	O17 - C20	+0.014112	+0.055000	+2.565047	-0.010274
22	N19 - C20	+0.355993	-0.686926	+0.176688	-1.026931
23	C20 - H21	+0.305435	-1.189628	+0.005468	-0.384076
24	C20 - C22	+0.286517	-0.749770	+0.125467	-0.341617
25	C22 - C23	+0.306794	-0.829336	+0.222838	-0.392064
26	C22 - C24	+0.303782	-0.798464	+0.186351	-0.390321
27	C23 - C25	+0.311212	-0.860509	+0.224681	-0.408972
28	C24 - H27	+0.289943	-1.036846	+0.012822	-0.356407
29	C24 - C26	+0.327463	-0.915063	+0.230564	-0.460300
30	C26 - C33	+0.238037	-0.526767	+0.030712	-0.237842

31	C33 - C54	+0.239177	-0.529808	+0.002659	-0.242200
32	C25 - C28	+0.304506	-0.780582	+0.220094	-0.397700
33	C26 - C28	+0.308086	-0.821354	+0.170587	-0.401454
34	C15 - O30	+0.241645	-0.421150	+0.036918	-0.525674
35	N18 - H29	+0.343246	-1.818020	+0.037073	-0.586412
36	H29 - F62	+0.103923	+0.370556	+0.032733	-0.156409
37	O30 - F62	+0.216821	+0.710127	+0.052328	-0.375318
38	H31 - F61	+0.105879	+0.146570	+0.006114	-0.132312
39	O30 - H31	+0.304463	-1.814718	+0.021042	-0.680613
40	C25 - C32	+0.242932	-0.548337	+0.031384	-0.248741
41	C33 - C34	+0.244009	-0.550633	+0.002457	-0.249822
42	C34 - H36	+0.286831	-0.994862	+0.001971	-0.359881
43	C33 - C38	+0.239588	-0.531281	+0.001868	-0.242482
44	C34 - H35	+0.289526	-1.020171	+0.002579	-0.365842
45	H27 - H36	+0.016899	+0.074250	+0.759255	-0.011513
46	C34 - H37	+0.285540	-0.988988	+0.002817	-0.355730
47	C38 - H40	+0.285245	-0.987242	+0.004326	-0.355058
48	C38 - H39	+0.287509	-1.005359	+0.004097	-0.359707
49	H41 - H58	+0.013787	+0.056128	+0.600833	-0.008630
50	C38 - H41	+0.287367	-0.999862	+0.003423	-0.361193
51	C32 - C42	+0.243938	-0.550806	+0.004931	-0.250924
52	C42 - H44	+0.288342	-1.014571	+0.003977	-0.359130
53	C42 - H43	+0.286044	-0.991734	+0.006649	-0.358495
54	H44 - O59	+0.009388	+0.036102	+0.346187	-0.006797
55	C42 - H45	+0.287815	-1.005496	+0.007444	-0.362516
56	C32 - C46	+0.241311	-0.538721	+0.005619	-0.246247
57	C23 - O59	+0.321050	+0.034937	+0.012558	-1.013323
58	C46 - H47	+0.284633	-0.979199	+0.013387	-0.355460
59	H48 - O59	+0.025059	+0.089138	+0.032475	-0.020774
60	C46 - H48	+0.294000	-1.065751	+0.005795	-0.367929
61	C46 - H49	+0.285058	-0.982196	+0.013201	-0.357687
62	C50 - H51	+0.287896	-1.007836	+0.002496	-0.361640
63	H53 - H58	+0.017807	+0.076497	+0.786464	-0.012251
64	C50 - H52	+0.286218	-0.995448	+0.002622	-0.356912
65	C32 - C50	+0.240009	-0.533451	+0.002137	-0.242508
66	C28 - H58	+0.285239	-1.006120	+0.010285	-0.341727
67	C50 - H53	+0.286177	-0.988718	+0.002008	-0.358873
68	C54 - H55	+0.285337	-0.987714	+0.005104	-0.355604
69	C54 - H56	+0.288775	-1.015645	+0.005079	-0.363111
70	C54 - H57	+0.285645	-0.990724	+0.004715	-0.355429
71	N19 - H60	+0.049501	+0.141469	+0.036608	-0.040771
72	O59 - H60	+0.356605	-2.310350	+0.012261	-0.740936
